# The role of electronic health records systems in de-implementing low-value care in primary care: a scoping review

**DOI:** 10.1186/s43058-025-00826-6

**Published:** 2025-12-19

**Authors:** Oliver T. Nguyen, Steven D. Vo, Dang Nguyen, Sri Varsha Katoju, Avaneesh R. Kunta, James H. Ford, Young-Rock Hong, Randa Perkins, Amir Alishahi Tabriz, Kea Turner

**Affiliations:** 1 Department of Health Outcomes and Behavior, H. Lee Moffitt Cancer Center & Research Institute, Tampa, FL USA; 2 Department of Industrial and Systems Engineering, University of Wisconsin at Madison, 1513 University Ave, Madison, WI 53706 USA; 3 Department of Epidemiology & Biostatistics, University of South Florida, Tampa, FL USA; 4 Corrigan Minehan Heart Center, Massachusetts General Hospital, Boston, MA USA; 5 Department of Community Health and Family Medicine, University of Florida, Gainesville, FL USA; 6 College of Medicine, University of Central Florida, Orlando, FL USA; 7 Division of Social and Administrative Sciences, School of Pharmacy, University of Wisconsin at Madison, Madison, WI USA; 8 Department of Health Services Research, Management, and Policy, University of Florida, Gainesville, FL USA; 9 Department of Internal Medicine, H. Lee Moffitt Cancer Center & Research Institute, Tampa, FL USA; 10 Department of Gastrointestinal Oncology, University of South Florida, Tampa, FL USA; 11 School of Nursing, University of North Carolina at Chapel Hill, Chapel Hill, NC USA

**Keywords:** Electronic health records, Low-value care, Implementation science, Primary care

## Abstract

**Background:**

Electronic health record (EHR) systems have been used to support the implementation of evidence-based care. Growing evidence suggests that EHR systems can also support de-implementation of low-value care. However, a review of this literature has not been conducted. This scoping review will: 1) summarize how EHR-based interventions themselves have been used in primary care settings to de-implement low-value care, 2) summarize the effectiveness of these EHR interventions, 3) describe de-implementation strategies and outcome measures that have been used, and 4) describe facilitators and barriers that influence EHR-based de-implementation interventions.

**Methods:**

We conducted a search using MEDLINE, CINAHL, Embase, and Web of Science on January 19, 2024 for peer-reviewed papers on EHRs and de-implementation in primary care. We inductively developed themes of how the EHR was used to support de-implementation. We mapped de-implementation strategies to a previously published taxonomy on implementation strategies, de-implementation outcomes to a previously published taxonomy on these outcomes, and facilitators and barriers to the Consolidated Framework for Implementation Research. We stratified study findings by EHR intervention type.

**Results:**

We included 50 studies. EHRs supported de-implementation using four intervention types: 1) EHR alerts, 2) order sets and preference lists, 3) documentation templates, and 4) communication tools among the care team. The proportion of studies that showed favorable effectiveness in reducing low-value care ranged from 16.7% (communication tools) to 50.0% (documentation templates). Common strategies to support EHR-based de-implementation interventions included auditing and providing feedback, conducting educational meetings, and distributing educational materials. Twenty-two studies reported some assessment of de-implementation outcomes. Most EHR intervention types had numerous multi-level facilitators and barriers identified.

**Conclusions:**

This scoping review identified multiple EHR-based interventions that health systems use to support de-implementation and their effectiveness. Although promising, the evidence base is limited by the general lack of frameworks used for intervention development and de-implementation, unclear theoretical rationale to support the use of selected de-implementation strategies, and the unclear validity of de-implementation outcomes used. Additional research is needed to develop and validate frameworks and outcomes for de-implementation to strengthen the evidence base.

**Trial registration:**

None.

**Supplementary Information:**

The online version contains supplementary material available at 10.1186/s43058-025-00826-6.

Contributions to the literature

- This scoping review identifies EHR tools that have been used to support de-implementation efforts.
- This scoping review attempts to summarize current attempts to measure de-implementation outcomes in the context of EHR de-implementation interventions.
- This review’s findings highlights a persistent need for the development of de-implementation frameworks, de-implementation outcome measures, and/or their operationalization into future research involving EHR de-implementation interventions.

## Introduction

The provision of primary care services, such as cancer screening and chronic care management, contributes to improved patient and health system outcomes. These include reduced risks of mortality [1–4], lower hospitalization rates [5–8], and reduced costs [2]. While primary care has multiple benefits, not all clinical practices rendered have resulted in improved outcomes, such as antibiotic prescribing for viral upper respiratory infections or ordering imaging for non-specific low back pain [9–17]. One review reported that the prevalence rates of many of these clinical practices have been moderately or very high [18]. For instance, the rate of antibiotic prescribing for viral upper respiratory infections can be as high as 89.0% [18]. Academics have described these types of practices as low-value care, which has been defined as “care that provides minimal or no health benefit” [19]. Unfortunately, the continued use of low-value care may have negative implications for health systems, including rising costs [20], patient harm [16, 17], and access issues (i.e., delaying or preventing patients from accessing services that are clinically efficacious for improving health outcomes) [21]. Consequently, the delivery of low-value care can also have adverse impacts on delivering primary care services efficiently especially as the demand for primary care exceeds the current supply of primary care practitioners [22]. Since primary care represents the gateway to the health care system, researchers have suggested that it can play a key role in limiting unnecessary healthcare use and interventions targeting the reduction of low-value care are particularly important in the primary care context [23].

A growing recognition from payers, health care quality researchers, and health system leaders exists to address low-value care by reinforcing the use of evidence-based care and de-implementing low-value care. One of the widely known attempts at addressing low-value care is the Choosing Wisely campaign, which had involved medical professional societies (e.g., American Academy of Family Physicians) [24] in identifying and disseminating information on what clinical practices were considered low-value care [25, 26]. Evaluations on the impact of this effort have found that this resulted in small changes in the use of low-value care [27, 28], suggesting a continued need to overcome known barriers to de-implementation (e.g., lack of awareness of alternative practices, forgetting to assess whether patient is ineligible/contraindicated to receive specific types of care) [29, 30]. There is an ongoing need to translate changes in clinical guidelines into meaningful changes in clinical practice. Using electronic health records (EHR) systems represents one promising approach to address some of the current barriers to de-implementing low-value care [31].

EHR systems have been widely adopted across many clinical settings [32], including primary care [33–35]. Since clinicians have to navigate through the EHR to perform many actions in their clinical workflows, such as placing orders and coordinating care among the team, EHRs have been used to support numerous quality improvement projects and other initiatives to increase the uptake of specific actions (e.g., clinical guideline adherence, identifying abnormal laboratory values) [36, 37]. Although the EHR’s ability to implement interventions and facilitate the uptake of clinical practices has been well demonstrated in the literature, to date, how it can promote the de-implementation of specific practices (e.g., low-value care) has not been fully explored. This information will be useful for helping guide health systems in optimally using the capabilities and available features of EHRs to support de-implementation efforts.

To address these gaps, this scoping review has four aims: 1) to summarize how EHR-based interventions have been used to de-implement low-value care within primary care settings, 2) to summarize the effectiveness of EHR-based interventions for de-implementing low-value care, 3) to identify what de-implementation strategies have been used and how de-implementation outcomes have been measured, and 4) to summarize the facilitators and barriers of the EHR-based de-implementation interventions. The findings may help inform health system leaders and informatics teams who are designing de-implementation interventions using EHR tools.

## Methods

We followed the Preferred Reporting Items for Systematic Reviews and Meta-Analyses (PRISMA) extension for scoping reviews (PRISMA-ScR) checklist [38]. We included the completed checklist in Additional File 1. This study was not registered in any review repository.

### Data sources and search

We developed a search strategy with a health sciences librarian that included subject headings and search terms for de-implementation, EHRs, and primary care settings. On January 19, 2024, we searched for all peer-reviewed literature in MEDLINE (PubMed), CINAHL, Embase, and Web of Science. No additional filters (e.g., years) were applied. Our complete search strategy is available in Additional File 2. We used Covidence to manage the scoping review process.

### Article screening and eligibility criteria

Our inclusion criteria included all articles that: 1) were written in English, 2) involved EHR features as part of the de-implementation intervention, 3) targeted a reduction in low-value care, and 4) involved only primary care settings or reported their data separately if other specialties were examined. We excluded articles that were non-empirical, were gray literature, did not involve the EHR or used only non-EHR interventions to de-implement a practice, did not report real-world data (e.g., model validation studies), or did not report intervention effectiveness, de-implementation outcomes, or facilitators and barriers. We did not restrict our search to studies that reported examining specific kinds of low-value care mentioned in clinical guidelines. Clinical guidelines generally advocate eliminating a specific practice. However, there are other de-implementation mechanisms, such as dis-investment. Thus, our approach allowed us to capture the range of de-implementation mechanisms that may have been used. Each article's title and abstract were independently screened for eligibility by any two reviewers of our review team (OTN, DN, SDV). For full-text review, each article was also independently screened by any two reviewers of our team (OTN, DN, SDV, SK, ARK). Conflicts were resolved by a third reviewer (KT).

### Data extraction

Two reviewers of our team (OTN, DN, SDV, SK, ARK) abstracted relevant data from included articles. We abstracted the following data from each included article: 1) country, 2) study design, 3) type of primary care setting, 4) low-value care targeted, 5) types of clinicians targeted, 6) theoretical frameworks used, 7) de-implementation strategies used, 8) types of de-implementation outcomes examined, 9) type of EHR intervention, 10) EHR vendor, 11) sample size, 12) major findings (intervention effectiveness and, if any, de-implementation outcomes), and 13) facilitators and barriers identified by study participants or the studies' authors. Conflicts were resolved by a third reviewer (KT).

### Data synthesis and presentation

We first inductively classified the types of EHR interventions that could be used to support de-implementation into four categories: 1) EHR alerts, 2) order sets and preference lists (i.e., bookmarked orders), 3) documentation templates, and 4) communication tools among the care team. These classifications were developed through the team’s consensus. EHR alerts included clinical decision support alerts, best practice advisory alerts, non-interruptive alerts, and EHR prompts. Order sets and preference lists encompassed EHR features that aimed to improve the efficiency of placing orders. Documentation templates included any pre-made textual data that clinicians could auto-populate in the note to save them from typing them. Communication tools included the use of tools, such as messaging and patient lists, that teams used to coordinate care.

We also classified low-value care using Verkerk et al.’s typology that suggests that this can be categorized as ineffective care, inefficient care, and unwanted care [19]. We separated our findings by each of the four EHR interventions when reporting on effectiveness, de-implementation strategies and outcomes, and facilitators and barriers. We followed the guidance proposed by Prusaczyk et al. that Proctors Implementation Outcomes framework could also be used to categorize the types of de-implementation outcomes as acceptability, appropriateness, adoption, costs, feasibility, fidelity, penetration, and sustainability [39].

Lastly, since we anticipated a diverse and broad range of facilitators and barriers among the included studies, we mapped facilitators and barriers that were either empirically identified or conjectured by the studies’ authors to the updated Consolidated Framework for Implementation Research (CFIR). The CFIR suggests that there are five levels of implementation factors (innovation, inner setting, outer setting, individuals, implementation process) [40]. Although the CFIR was designed for use with the implementation of interventions, it has also been successfully applied to studying de-implementation interventions [30]. Since there would be greater value in reporting facilitators and barriers that could be more directly traced back to whether a de-implementation effort was successful or not, we only reported facilitators and barriers in this manuscript if the study reported whether the intervention was effective or not. For studies that only reported qualitative findings, we searched authors’ bibliographies to see if they had published another study on the same intervention before determining whether or not to include the qualitative study’s facilitators and barriers in our analysis. All data synthesis activities were done through consensus of the study team.

## Results

### Study characteristics

**Fig. 1:**
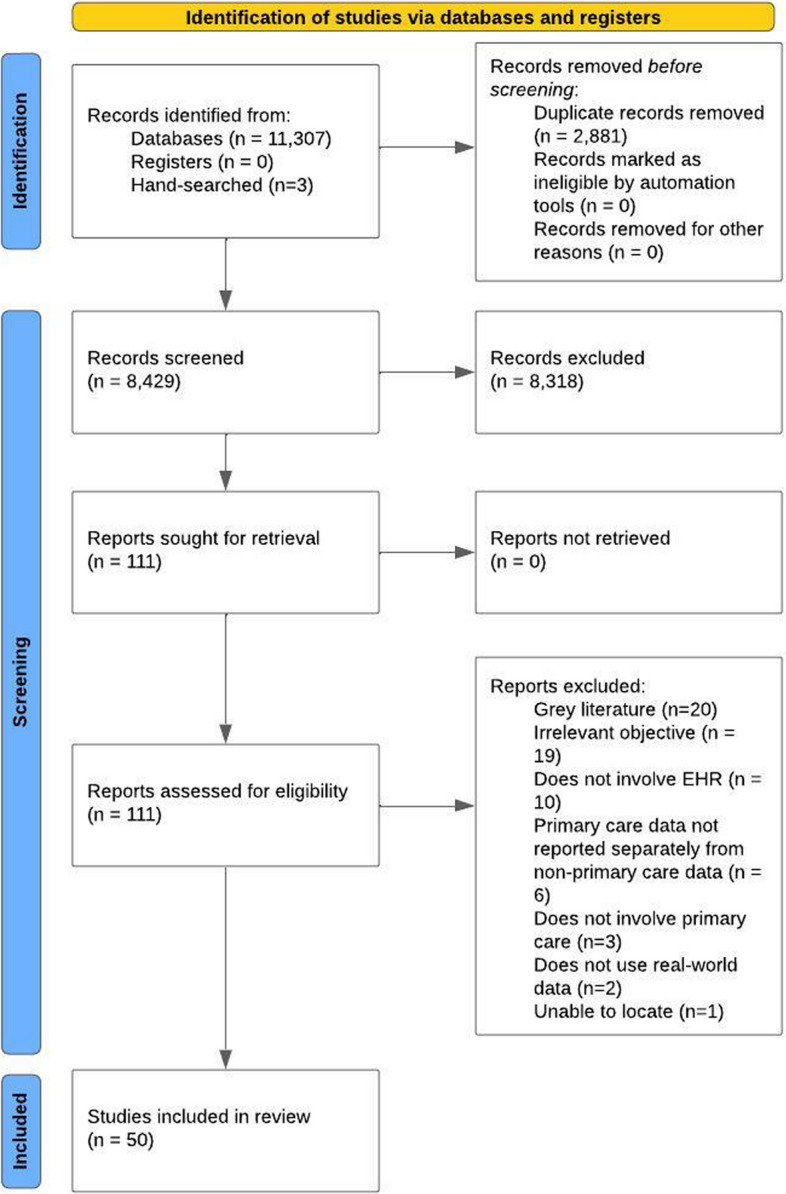
PRISMA Flowchart

After title, abstract, and full-text reviews, we included 50 articles (Fig. 1). Most studies occurred in the United States (*n* = 34, 68.0%) or Canada (*n* = 7, 14.0%). Quasi-experimental study designs were commonly used (*n* = 30, 60.0%). Of the three types of low-value care, only ineffective and inefficient care had been studied. Ineffective care was examined in most of the studies (*n* = 34, 68.0%). Almost half of the studies (*n* = 15) on ineffective care focused on antibiotic prescribing. Inefficient care was more diverse and generally focused on diagnostic orders (13/16, 81.3%). Most studies (*n* = 44, 88.0%) did not report the use of a conceptual framework. In the remaining studies, the few frameworks that were used that come from implementation science included PRECEDE-PROCEED (*n* = 2), RE-AIM (*n* = 1), and knowledge-to-action (*n* = 1). Half of the included studies reported the name of the EHR vendor used. Of these studies, most used Epic (*n* = 13). We were able to identify who the EHR intervention targeted in 39 (78.0%) of the studies. Physicians were commonly targeted (*n* = 38) followed by nurse practitioners (*n* = 19) and physician assistants (*n* = 12). Pharmacists (*n* = 1) and nurses were rarely targeted (*n* = 3) (Additional File 3). EHR alerts were the most commonly studied EHR intervention (*n* = 28, 56.0%) (Table 1).

**Table 1:** Summary table of effectiveness per EHR intervention type

EHR Intervention Type	Total # of Studies ^a^	# of Studies Reporting Favorable Effectiveness n (%)	# of Studies Reporting Mixed Effectiveness n (%)	# of Studies Reporting Null Effectiveness n (%)	# of Studies Reporting Worsened Effectiveness n (%)	# of Studies Without Significance Testing or No Quantitative Results n (%)
EHR Alerts	28	12 (42.9)	5 (17.9)	5 (17.9)	0 (0.0)	6 (21.4)
Order Sets and Preference Lists	13	5 (38.5)	3 (23.1)	3 (23.1)	0 (0.0)	2 (15.4)
Documentation Templates	6	3 (50.0)	2 (33.3)	0 (0.0)	0 (0.0)	1 (16.7)
Communication Tools Among the Care Team	6	1 (16.7)	0 (0.0)	1 (16.7)	0 (0.0)	4 (66.7)

^a^A few studies examined more than one EHR intervention type

### Intervention effectiveness

Table 1 is a summary table of whether the studies reported favorable, mixed, null, or worsened effectiveness or whether the study did not conduct significance testing or were only reporting qualitative findings. Additional File 4 presents specific findings for each of the four EHR interventions. We summarize those findings below.

#### EHR alerts

Twenty-eight studies evaluated the use of EHR alerts to de-implement a practice [41–68]. Twelve of these studies showed favorable findings, such as reducing inappropriate tests [41, 43, 49, 51, 52], reducing inappropriate antibiotic prescribing [46–48], and reducing inappropriate imaging [50].

#### Order sets and preference lists

Thirteen studies evaluated the use of order sets and preference lists to de-implement a practice [60, 69–80]. Five of these studies showed favorable findings, such as reducing inappropriate antibiotic prescribing and reducing unnecessary tests [69, 71].

#### Documentation templates

Six studies evaluated the use of documentation templates to de-implement a practice [71, 81–85]. Half of the studies reported favorable findings, such as improving appropriate antibiotic prescribing [71], discontinuing proton pump inhibitors [81], and reducing the dosage of morphine [82].

#### Communication tools among the care team

Six studies evaluated the use of a communication tool in the EHR to coordinate care or use a team-based approach to de-implement a practice [79, 86–90]. Although all studies reported point estimates, most did not conduct statistical significance testing. Only one study has reported favorable findings, which saw higher odds of discontinuing opioid treatments and reducing opioid dose amounts by at least 10% by coordinating a team-based approach in the EHR [86].

### De-implementation strategies used

Across the studies, the most common high-level groupings of de-implementation strategies used were supporting clinicians and changing infrastructure. Given the focus of this scoping review, the most common de-implementation strategies were unsurprisingly creating reminders for clinicians and changing record systems. Beyond this, other common strategies included auditing and providing feedback, conducting educational meetings, and distributing educational materials. The number of de-implementation strategies used ranged from one to nine, with EHR alerts using an average of 4.68 strategies, order sets and preference lists using an average of 3.46 strategies, documentation templates using an average of 5.67 strategies, and communications tools using an average of 3.50 strategies. A summary of the de-implementation strategies used across the studies for each EHR intervention is displayed in Table 2. Specific implementation strategies used for each study are available in Additional File 5.

**Table 2:** De-implementation strategies used per EHR intervention type

EHR Intervention Type	Use Evaluative and Iterative Strategies	Provide Interactive Assistance	Adapt and Tailor to the Context	Develop Stakeholder Interrelationships	Train and Educate Stakeholders	Support Clinicians	Engage Consumers	Utilize Financial Strategies	Change Infrastructure	Citations
EHR Alerts	Assess for readiness and identify barriers and facilitators, audit and provide feedback, conduct local needs assessment, obtain and use patients/consumers and family feedback, stage implementation	Centralize technical assistance	Tailor strategies	Develop academic partnerships, identify and prepare champions, involve executive boards, organize clinician implementation team meetings, use advisory boards and workgroups	Conduct educational meetings, conduct educational outreach visits, conduct ongoing training, develop educational materials, distribute educational materials, use train-the-trainer strategies, work with educational institutions	Facilitate relay of clinical data to providers, remind clinicians	Intervene with patients/consumers to enhance uptake and adherence, involve patients/consumers and family members	Alter incentive/allowance structures, alter patient/consumer fees	Change record systems	Ackerman et al., 2013 [63]; Alagiakrishnan et al., 2016 [64]; Alagiakrishnan et al., 2019 [58]; Anderson et al., 2020 [41]; Campbell et al., 2021 [59]; Cole et al., 2020 [42]; Delvaux et al., 2020 [43]; Feldstein et al., 2006 [44]; Fried et al., 2017 [45]; Gill et al., 2011 [53]; Gonzales et al., 2013 [46]; Gulliford et al., 2019 [47]; Hingorani et al., 2015 [48]; Howell et al., 2014 [49]; Ip et al., 2014 [50]; Keohane et al., 2017 [65]; Mann et al., 2020 [60]; McDermott et al., 2014 [66]; Meeker et al., 2016 [54]; Persell et al., 2016 [61]; Petrilli et al., 2018 [51]; Rowe et al., 2023 [62]; Shelton et al., 2015 [52]; Singhal et al., 2022 [67]; Tamblyn et al., 2003 [55]; Tamblyn et al., 2008 [56]; Walsh et al., 2016 [68]; Wessell et al., 2013 [57]
Order Sets and Preference Lists	Audit and provide feedback, conduct local needs assessment	None	Tailor strategies	Conduct local consensus discussions, Involve executive boards	Conduct educational meetings, conduct ongoing training, develop educational materials, distribute educational materials, work with educational institutions	Create new clinical teams, remind clinicians	Involve patients/consumers and family members	None	Change record systems	Ancker et al., 2021 [77]; Buehrle et al., 2020 [69]; Khadadah et al., 2022 [75]; Lin et al., 2020 [78]; Mann et al., 2020 [60]; Martins et al., 2017 [76]; Matulis et al., 2017 [70]; McCormick et al., 2020 [71]; Rozario et al., 2020 [72]; Seppänen et al., 2016 [73]; Singh-Franco et al., 2022 [79]; van Wijk et al., 2001 [74]; Vardy et al., 2005 [80]
Documentation Templates	Assess for readiness and identify barriers and facilitators, audit and provide feedback, conduct local needs assessment	None	None	Identify and prepare champions, organize clinician implementation team meetings	Conduct educational meetings, conduct educational outreach visits, develop educational materials, distribute educational materials	Remind clinicians	Involve patients/consumers and family members	None	Change record systems, mandate change	Litvin et al., 2012 [84]; Litvin et al., 2013 [83]; Mainous et al., 2013 [85]; McCormick et al., 2020 [71]; Nallapeta et al., 2020 [81]; Wong et al., 2019 [82]
Communication Tools Among the Care Team	Stage implementation	None	Tailor strategies	Use advisory boards and workgroups, identify and prepare champions	Conduct educational meetings, distribute educational materials	Revise professional roles, remind clinicians	Involve patients/consumers and family members	None	Change record systems	Cossette et al., 2019 [88]; Lagisetty et al., 2020 [87]; Liebschutz et al., 2017 [86]; Milone et al., 2014 [89]; Odenthal et al., 2020 [90]; Singh-Franco et al., 2022 [79]

### De-implementation outcomes assessed

More than half (*n* = 22, 44.0%) of the included studies reported some assessment of de-implementation outcomes. The prevalence of these assessments differed based on EHR intervention. We found assessments of de-implementation outcomes in 50.0% of studies on EHR alerts, documentation templates, and communication tools within the care team. However, only 23.1% of studies on order sets and preference lists reported assessments on de-implementation outcomes. (Table 3).

**Table 3:** Assessment of de-implementation outcomes among included studies

Study	Acceptability	Adoption	Appropriateness	Costs	Feasibility	Fidelity	Penetration	Sustainability
Ackerman 2013 [63]	Yes	Yes	Yes	No	Yes	No	No	No
Alagiakrishnan 2016 [64]	Yes	Yes	Yes	No	Yes	Yes	No	No
Alagiakrishnan 2019 [58]	No	No	No	No	No	No	No	No
Ancker 2021 [77]	No	No	No	No	No	No	No	No
Anderson 2020 [41]	No	No	No	Yes	No	No	No	No
Buehrle 2020 [69]	No	No	No	No	No	No	No	No
Campbell 2021 [59]	No	No	No	No	No	Yes	No	No
Cole 2020 [42]	No	No	No	No	No	No	No	No
Cossette 2019 [88]	Yes	No	Yes	No	No	No	No	No
Delvaux 2020 [43]	No	No	No	No	No	No	No	No
Feldstein 2006 [44]	No	No	No	No	No	No	No	No
Fried 2017 [45]	No	No	No	No	No	No	No	No
Gill 2011 [53]	Yes	Yes	No	No	Yes	Yes	No	No
Gonzales 2013 [46]	No	No	No	No	No	No	Yes	No
Gulliford 2019 [47]	Yes	Yes	No	Yes	Yes	Yes	Yes	No
Hingorani 2015 [48]	No	No	No	No	No	No	Yes	No
Howell 2014 [49]	No	No	No	No	No	No	No	No
Ip 2014 [50]	No	No	No	No	No	No	No	No
Keohane 2017 [65]	No	No	No	No	No	No	No	No
Khadadah 2022 [75]	No	No	No	Yes	No	No	No	No
Lagisetty 2020 [87]	Yes	No	Yes	No	Yes	No	No	Yes
Liebschutz 2017 [86]	No	No	No	No	No	No	No	Yes
Lin 2020 [78]	No	No	No	No	No	No	No	No
Litvin 2012 [84]	No	Yes	Yes	No	Yes	Yes	Yes	No
Litvin 2013 [83]	No	No	Yes	No	Yes	No	No	No
Mainous 2013 [85]	No	Yes	No	No	No	No	Yes	No
Mann 2020 [60]	No	Yes	No	No	No	Yes	Yes	Yes
Martins 2017 [76]	No	No	No	No	No	No	No	No
Matulis 2017 [70]	No	No	No	No	No	No	No	No
McCormick 2020 [71]	No	No	No	No	No	No	No	No
McDermott 2014 [66]	Yes	No	Yes	No	Yes	Yes	No	No
Meeker 2016 [54]	No	No	No	No	No	No	No	No
Milone 2014 [89]	No	No	No	No	No	No	No	No
Nallapeta 2020 [81]	No	No	No	No	No	No	No	No
Odenthal 2020 [90]	No	No	No	No	No	No	No	No
Persell 2016 [61]	No	Yes	No	No	No	No	No	No
Petrilli 2018 [51]	No	No	No	No	No	No	No	No
Rowe 2023 [62]	Yes	No	Yes	No	Yes	Yes	No	No
Rozario 2020 [72]	No	No	No	Yes	No	No	No	No
Seppänen 2016 [73]	No	No	No	No	No	No	No	No
Shelton 2015 [52]	No	No	No	No	No	No	No	No
Singh-Franco 2022 [79]	No	No	No	No	No	No	No	No
Singhal 2022 [67]	No	No	No	No	No	No	No	No
Tamblyn 2003 [55]	No	Yes	No	No	No	No	No	No
Tamblyn 2008 [56]	No	No	No	No	No	No	No	No
vanWijk 2001 [74]	No	No	No	No	No	No	No	No
Vardy 2005 [80]	No	No	No	No	No	No	No	No
Walsh 2016 [68]	Yes	No	No	No	Yes	No	Yes	No
Wessell 2013 [57]	No	No	No	No	No	No	No	No
Wong 2019 [82]	No	No	No	No	No	No	No	No
Total	9	9	8	4	10	8	7	3

Across all included studies, each of the eight de-implementation outcomes were assessed. From most frequent to least, these included feasibility (*n* = 10, 20.0%), acceptability (*n* = 9, 18.0%), adoption (*n* = 9, 18.0%), appropriateness (*n* = 8, 16.0%), fidelity (*n* = 8, 16.0%), penetration (*n* = 7, 14.0%), costs (*n* = 4, 8.0%), and sustainability (*n* = 3, 6.0%) (Table 3). The four EHR intervention types also differed in the outcomes assessed. Across studies using EHR alerts, all eight outcomes were assessed. Among studies examining order sets and preference lists, only costs and fidelity had been assessed. For studies using documentation templates, all outcomes had been assessed except costs and sustainability. Lastly, across studies using communication tools among the care team, only acceptability, appropriateness, feasibility, and sustainability had been assessed (Additional File 6).

Measurement approaches also varied by outcome type. Feasibility assessments used surveys or semi-structured interviews. Acceptability assessments used surveys, semi-structured interviews, and informally collected feedback from participants. Adoption assessments used surveys, semi-structured interviews, and authors’ observations from tracking which participant/site agreed to participate in the study. Appropriateness assessments used surveys, semi-structured interviews, anecdotal feedback, and tracked whether clinicians thought alerts were clinically relevant. Cost assessments usually aimed to proxy the cost savings to the health system after de-implementing the practice. The data sources for these included internal costs to deliver a health care service, reimbursement rates that payers would have paid for the de-implemented practice, and actual reimbursement rates that have been paid to the health system after contract negotiations. Most studies did not describe what was involved in developing and rolling out the EHR de-implementation intervention. Feasibility assessments used semi-structured interviews and anecdotal feedback. Fidelity assessments used surveys, semi-structured interviews, and system logs. Penetration assessments both used surveys and system logs. Lastly, sustainability assessments used semi-structured interviews, authors’ observations on how sites responded to initial pilot study findings, and system logs (Additional File 6).

A brief summary of the actual findings from the de-implementation outcomes for each EHR intervention type is as follows. Specific findings are available in Additional File 6.

#### EHR alerts

Cost savings were estimated to be $51,538 cost savings in a six-month period and differences may not exist between intervention and control groups [41]. Mixed perceptions of feasibility existed. Some clinicians reported the ability to integrate the alerts into clinical practice, easy to use, and that the alerts were not time-consuming [66]. Other clinicians felt there was not enough time to read the alerts, did not notice the alerts, had too much information, did not provide explicit details on how to discontinue drugs or prescribe alternate drugs, or hesitated to discontinue other clinicians’ orders [53, 62–64]. Fidelity issues may exist since studies reported that the tools were not used as expected during eligible visits or that the alerts were presented or did not fire based on missing or outdated EHR data [59]. Penetration was mild to moderate with no study reporting a rate of over 51% [46–48, 53]. Sustainability issues may occur since the use of tools in the alerts (e.g., calculators) decreased over time [60].

#### Order sets and preference lists

Cost savings were highly varied, ranging from $124,380 CAD (~ $90,016.29 USD) to $1,060,640 USD per year [72, 75]. Fidelity appeared to be low in the only study that assessed this where less than 2% of order sets were accepted by clinicians during visits [60].

#### Documentation templates

Clinicians generally found templates acceptable [84]. Adoption typically was high [84]. Appropriateness issues emerged whenever clinicians disagreed with the merits of a prescribing guideline or felt that the guidelines were not applicable to specific types of patients [84]. Although clinicians generally found templates feasible in deprescribing, they also felt that inability to modify the template themselves, lack of technical support, fear of missing diagnoses, and inability to directly address patients who demanded medications were current feasibility issues [84]. Fidelity challenges could occur if there are no computers in the exam room, forcing the clinician to use the template before or after the visit rather than during the visit as intended. Penetration estimates were highly varied, ranging from 0 to 77% [84, 85].

#### Communication tools among the care team

Overall, clinicians seemed to find the involvement of the care team in deprescribing initiatives highly acceptable [87]. Although appropriateness was generally high, clinicians raised concerns related to appropriateness when working with patients who already have poor relationships with the clinician or would become distrustful towards seeing anyone else on the care team [87]. Clinicians seemed to perceive interventions as feasible due to, in part, the use of similar intervention components in other areas of primary care (e.g., chronic care management) [87]. Sustainability issues identified included whether pharmacist staffing levels could be maintained and the reliance on hiring additional nurses to continue use of the interventions [86, 87].

### De-Implementation facilitators and barriers

Of the included studies, 33 (66.0%) reported potential facilitators and barriers to EHR de-implementation interventions. Table 4 displays the facilitators and barriers per EHR intervention type. We summarize these below.

**Table 4:** Facilitators and barriers per EHR intervention type

EHR Intervention Type	Domain	Facilitators	Barriers	Citations
EHR Alerts	Innovation	Forced clinicians to reflect on the rationale of prescribing decision, could serve as education or reminders for the clinician, facilitated patient education on medication safety; tailoring the innovation to integrate into varied workflows, usefulness of prompts, including deprescribing tools within the alert, alert specificity, use of structured clinical data for tool	Usability issues, alert did not provide more guidance on safer alternatives or information on how to monitor side effects, algorithm makes incorrect recommendations due to missing data; alerts did not fire appropriately due to missing data in the EHR; perceived disruptiveness to workflow; alerts were not clinically relevant, low saliency of alerts, using non-interruptive alerts, some clinicians bypassed the alerts by having medical assistants begin the encounter	Ackerman et al., 2013 [63]; Alagiakrishnan et al., 2016 [64]; Anderson et al., 2020 [41]; Campbell et al., 2021 [59]; Delvaux et al., 2020 [43]; Gill et al., 2011 [53]; Gulliford et al., 2019 [47]; McDermott et al., 2014 [66]; Mann et al., 2020 [60]; Shelton et al., 2015 [52]; Tamblyn et al., 2008 [56]; Walsh et al., 2016 [68]
	Outer Setting	No findings available	Medical liability structures	Ackerman et al., 2013 [63]
	Inner Setting	Personal workflows were also revised	Insufficient time during patient visit to provide patient education, not using the EHR at the point of care; clicking past alerts without reading them due to alert fatigue, deprescribing is not part of the culture; lack of financial incentives or personal recognition; complexity of EHR workflows, computer issues	Ackerman et al., 2013 [63]; Alagiakrishnan et al., 2016 [64]; Anderson et al., 2020 [41]; Campbell et al., 2021 [59]; Delvaux et al., 2020 [43]; Gill et al., 2011 [53]; Gulliford et al., 2019 [47]; Mann et al., 2020 [60]; McDermott et al., 2014 [66]; Tamblyn et al., 2003 [55]; Walsh et al., 2016 [68]
	Individuals	Younger patient age	Diagnostic uncertainty, clinicians' beliefs that published studies are not generalizable to their patients, clinicians' beliefs that patient education would not be effective, clinicians' beliefs that they do not personally overprescribe; hesitation to discontinue orders made by other clinicians; disagreement with clinical guidelines; clinicians' overconfidence in their diagnoses, skepticism towards the existence on consequences for overprescribing, clinicians already thought they were following guidelines, belief that patients can tolerate medications and any side effects, concerns that patient will have withdrawal symptoms if medication was deprescribed, concerns that alert guidance could threaten relationship with patient, patients expect treatment (e.g., antibiotics), patients' comorbidities	Ackerman et al., 2013 [63]; Alagiakrishnan et al., 2016 [64]; Cole et al., 2020 [42]; Gill et al., 2011 [53]; Gulliford et al., 2019 [47]; Howell et al., 2014 [49]; Mann et al., 2020 [60]; McDermott et al., 2014 [66]; Singhal et al., 2022 [67]; Tamblyn et al., 2008 [56]; Walsh et al., 2016 [68]
	De-implementation Process	Patient education materials, physician education, use of clinical champions and EHR support team, raising awareness of the tool's existence	Patient education materials were not perceived by clinicians as effective, using external IT staff to implement innovation	Ackerman et al., 2013 [63]; Gonzales et al., 2013 [46]; Gulliford et al., 2019 [47]; Howell et al., 2014 [49]; McDermott et al., 2014 [66]
Order Sets and Preference Lists	Innovation	Making ordering inappropriate tests more tedious	Clinicians could bypass the order set's recommendations, alerts were not clinically relevant, no hard stops, no point-of-care education provided to clinician	Buehrle et al., 2020 [69]; Mann et al., 2020 [60]; Rozario et al., 2020 [72]; Seppänen et al., 2016 [73]; Singh-Franco et al., 2022 [79]
	Outer Setting	No findings available	No findings available	
	Inner Setting	Guideline adherence rate was low prior to innovation's implementation	Templated orders were not part of the culture, time pressure, alert fatigue, complexity of EHR workflows, clinician could still place order inappropriately from preference lists or manually searching the order	Ancker et al., 2021 [77]; Matulis et al., 2017 [70]; Mann et al., 2020 [60]; Rozario et al., 2020 [72]; Singh-Franco et al., 2022 [79]
	Individuals	Willingness to embrace clinical guidelines	Diagnostic uncertainty, clinicians' overconfidence in their diagnoses; skepticism towards the existence on consequences for overprescribing, patients expect treatment (e.g., antibiotics)	Lin et al., 2020 [78]; Mann et al., 2020 [60]
	De-implementation Process	Peer comparisons	Not having IT support, no usability feedback channels available	Buehrle et al., 2020 [69]; Matulis et al., 2017 [70]
Documentation Templates	Innovation	Facilitated patient discussions, providing patient education content in template, minimal disruptiveness to workflows, serves as reminders	Disruptive to workflows	Litvin et al., 2012 [84]; Litvin et al., 2013 [83]
	Outer Setting	No findings available	Difficulty hiring clinical staff members	Nallapeta et al., 2020 [81]
	Inner Setting	No findings available	Higher comfort and preferences for template versions prior to the innovation's changes, challenges in using template when patients have multiple chief complaints, computer issues, computers not located in exam room, limited resources for printing patient education materials, time constraints	Litvin et al., 2012 [84]; Nallapeta et al., 2020 [81]
	Individuals	Personal commitment to use guidelines, agreement with guidelines, perception that tool increases awareness of prescribing guidelines, perception that tool facilitates visit efficiency	Disagreement with clinical guidelines in template, concerns on missing serious diagnoses; diagnosing patients differently to bypass the tool; patients expect treatment (e.g., antibiotics)	Litvin et al., 2012 [84]; Litvin et al., 2013 [83]; Mainous et al., 2013 [85]
	De-implementation Process	Technical support, providing training, having non-physicians of care team initiate the template, using performance reports to support innovation, having team huddles, clinician involvement in decision-making	Training was required	Litvin et al., 2012 [84]; Litvin et al., 2013 [83]; Nallapeta et al., 2020 [81]; Wong et al., 2019 [82]
Communication Tools Among the Care Team	Innovation	No findings available	No findings available	
	Outer Setting	No findings available	Difficulty hiring clinical staff members	Lagisetty et al., 2020 [87]
	Inner Setting	No findings available	No findings available	
	Individuals	No findings available	No findings available	
	De-implementation Process	Use of nurse care managers, having a written tapering schedule, patient education by pharmacist, pharmacists followed up with patients after visit	No findings available	Liebschutz et al., 2017 [86]; Odenthal et al., 2020 [90]

#### EHR alerts

Facilitators spanned the innovation-level (e.g., integrating deprescribing tools within the alert, alert specificity), inner setting (workflows), and process-level (e.g., providing patient education as well). Barriers spanned all five levels, such as innovation-level (usability issues, inadequate guidance on safer alternatives), outer setting level (e.g., medical liability structures), inner setting level (e.g., insufficient time in the visit to provide patient education, not using the EHR during the visit), and individual characteristics (e.g., patient expectations for treatment).

#### Order sets and preference lists

Facilitators spanned the innovation-level (making ordering inappropriate tests more tedious, inner setting level (low baseline guideline-adherence rates), individuals (willingness to use clinical guidelines), and process (using peer comparisons). Barriers spanned all five levels, such as the innovation-level (e.g., ability to bypass order set’s recommendations, no hard stops), inner setting level (e.g., templated orders were not part of the culture, alert fatigue), and individual-level (e.g., skepticism towards negative consequences of overprescribing).

#### Documentation templates

Facilitators spanned the innovation-level (e.g., providing patient education in template, having reminders), individual-level (e.g., agreement with guidelines, perception the template facilitates visit efficiency), and process-level (e.g., having non-physicians initiate the template). Barriers spanned all five levels, such as the innovation-level (disruptive to workflows), outer setting level (e.g., patients’ expectations for treatment even if not clinically indicated), and individual-level (e.g., disagreement with guidelines, fear of missing a diagnosis).

#### Communication tools among the care team

Facilitators were process-related and mostly focused on the availability of care team members while the only barrier reported so far was outer setting-related and involved difficulty of hiring staff.

## Discussion

### Primary findings

This scoping review aimed to summarize the available evidence on how EHRs have been used to de-implement low-value care in primary care settings. Our secondary aims were to summarize the interventions’ effectiveness, de-implementation outcomes used, and facilitators and barriers of these interventions. Overall, most EHR-based interventions involved the use of clinical decision support or best practice alerts with fewer interventions that used the EHR to modify order sets, provide documentation templates, or as a communication tool among the care team (e.g., pharmacist to the physician). The use of de-implementation strategies and the measurement of de-implementation outcomes as well as the identification of facilitators and barriers varied depending on the EHR intervention type. We discuss the implications for research below (Table 5).

**Table 5:** Summary of research implications

EHR Intervention Type	Evidence Gaps	Research Implications
EHR Alerts	- Incorrect data in the EHR lead to incorrectly alerting clinicians to de-implement a clinical practice - EHR alerts by themselves appear to result in mixed impact on de-implementation among some clinicians, partly due to the belief that de-implementation guidance does not apply to them	- Explore to what extent strategies currently used when using EHR alerts (e.g., preserving clinician autonomy while presenting clinical guidelines, improving alert usability) to facilitate uptake of clinical guidelines extend to using EHR alerts to support de-implementation
Order Sets and Preference Lists	- Unclear why acceptance rates of order sets aiming to de-implement care are low	- Explore the acceptability, appropriateness, and feasibility of using order sets and preference lists to support de-implementation efforts
Documentation Templates	- Unclear how templates support de-implementation initiatives	- Explore how template use or how changes to templates by clinicians influence de-implementation - Assess whether combining templates (a passive form of influencing change) with more active EHR intervention types (e.g., EHR alerts, order sets) leads to differences in de-implementation outcomes and effectiveness
Communication Tools Among the Care Team	- Paucity of research in how the EHR supports communication and coordination of de-implementation initiatives	- Additional studies that assess a wider range of de-implementation outcomes and effectiveness of these tools are needed
General Research Needs	- Most studies did not report a conceptual framework	- Disseminate existing de-implementation frameworks to improve awareness - Assess to what extent that technology acceptance frameworks to understand the intention to use the EHR to de-implement a clinical practice
	- It is unclear how design elements of EHR-based de-implementation interventions influence de-implementation and effectiveness outcomes	- Explore how different nudging techniques influence de-implementation
	- To our knowledge, no validated de-implementation measures exist	- Assess to what extent current implementation measures retain psychometric properties when used to evaluate de-implementation - Develop specific de-implementation measures and test them for psychometric strength
	- Costs of de-implementation remain unclear	- Develop cost assessment instruments with psychometric properties to standardize the reporting of de-implementation costs
	- No studies de-implemented unwanted care	- Explore strategies that are used to de-implement unwanted care to see if they differ from those used to de-implement ineffective or inefficient care
	- Unclear whether other EHR interventions can also be used to support de-implementation	- Assess how other EHR interventions, such as patient dashboards and patient portals, can support de-implementation efforts
	- Unclear whether any EHR intervention identified in this review is stronger at influencing de-implementation than others	- Conduct comparative effectiveness in de-implementation among various EHR interventions
	- Unclear how EHR interventions can support de-implementation across the care team	- Examine if de-implementation outcomes and effectiveness vary between physicians and other members of the care team (e.g., physician assistants, nurse practitioners, pharmacists, dietitians, psychologists)

### EHR intervention types

EHR alerts were the most common intervention type to de-implement low-value care. Although EHR alerts represented the intervention type with the largest percentage of studies demonstrating their effectiveness, our review identified mixed perceptions of acceptability, appropriateness, and feasibility. Issues with fidelity, penetration, and sustainability were also commonly reported. Some of the concerns raised included the alerts firing or not firing due to the use of outdated or missing data and perceived threats to clinical autonomy. Additional barriers, such as usability issues and clinicians’ perceptions that the alerts and guidance do not apply to them or their patients, were also identified. These findings highlight multi-level issues that may inhibit long-term success of relying on EHR alerts for de-implementation and likely will require targeted strategies to address usability, clinician engagement, workflow integration, and ongoing monitoring and adaptation to ensure sustained effectiveness.

Order sets and preference lists both help reduce the number of steps needed to place orders, reducing some barriers to complying with guidelines [91]. Our review suggests that these tools have the potential to support de-implementation efforts and may contribute to cost savings for the health system. However, one concern we found was the low acceptance rates of the default order sets (i.e., not changing the orders suggested), limited evaluations on de-implementation outcomes, and clinicians’ ability to bypass the order set’s recommendations. These findings suggest a need for additional evaluations that evaluate de-implementation outcomes (e.g., acceptability, appropriateness, feasibility) to better understand how and to what extent changes to order sets and preference lists can support de-implementation efforts. This information can help health systems modify and/or retire underperforming order sets [92, 93].

Documentation templates have generally experienced high uptake and they contribute to care in several ways, such as serving as reminders to the clinicians [94]. In this review, although there were a limited number of studies evaluating documentation templates, half of the studies reported favorable findings with the other half reporting mixed findings. No study reported worsened outcomes or null associations. However, we found that although studies initially reported high intention to use templates, the actual use was lower. The combined findings suggest that documentation templates can facilitate de-implementation in specific contexts (e.g., patient is insistent on receiving antibiotics even when not clinically indicated). Accordingly, it may be valuable to understand what contexts support the use of templates for de-implementation. Since templates are known to be modified by clinicians to include personalized unstructured text or to remove irrelevant text [95], it may also be valuable to further study how templates are modified. To our knowledge, while there is growing interest in using EHR logs and vendor-derived EHR use measures to monitor the use of templates and other features [96, 97], no study has yet tracked how often and what changes are made to templates. To date, EHR logs and vendor-derived EHR use measures can determine if templates were used, who initiated them, and when that occurred [96, 97]. Knowing how templates are changed could be valuable in the evaluation of templates in quality improvement work. We also noted that, compared to EHR alerts and order sets and preference lists, documentation templates may be a relatively more passive form of influencing changes in clinician behavior. Future research should examine whether the use of templates synergizes with the use of any of the active forms of EHR interventions. Multi-level strategies that also target patients may also be necessary if they are known to contribute to the targeted low-value practice.

Care team models in primary care have been shown to contribute to favorable patient and process outcomes, such as improved continuity of care and improved mental health outcomes [98, 99]. However, achieving these requires the coordination of tasks and care. To address the increasing complexity of care and care coordination needs, we found a few studies evaluating communication tools in the EHR to support de-implementation using a team approach. A limited number of studies evaluated their use in de-implementation efforts with even fewer that quantitatively assessed the effectiveness of these tools. Of the two studies that did [86, 87], one reported favorable findings [86]. To date, although not every type of de-implementation outcome has been examined, existing studies seem to suggest favorable acceptability, appropriateness, and feasibility. Insufficient evidence to recommend their use in future de-implementation efforts exists. However, current findings are encouraging and suggest a need for additional research that formally tests their impact using more rigorous study designs (e.g., using comparison groups, significance testing). The general focus of the identified facilitators and barriers coupled with the extant findings on sustainability and the use of the revision of professional roles as an implementation strategy also point toward the need of developing staffing models in primary care when using integrated care teams.

### Other implications for research

We highlight several implications for researchers moving forward. First, most studies did not report the use of a theory or framework to inform the intervention design, selection of de-implementation strategies, or de-implementation outcomes used. This finding echoes those found in other studies [100–103]. This trend may be, in part, due to the relatively low number of de-implementation frameworks available in contrast to the number of implementation frameworks [104, 105]. Interestingly, to our knowledge, there is at least one de-implementation framework developed for use in primary care settings [106]. Unfortunately, the overall underuse of theories and frameworks in the included studies likely contributed to the sparse details of how de-implementation strategies were operationalized and how the selection of de-implementation outcomes were selected and assessed. Researchers may benefit from less complex frameworks to improve their operationalization into studies and from dissemination of these frameworks through the web, such as the web tool on dissemination and implementation frameworks by the University of Colorado Denver [107], to improve awareness.

Second, technology acceptance frameworks, such as the Technology Acceptance Model or the Unified Theory of Acceptance and Use of Technology theory [108, 109], represent another type of framework that could be useful in building EHR-based de-implementation interventions and studying whether they influence clinicians’ behaviors, such as whether they increase the uptake of an EHR feature (e.g., dose calculator) or replace a given EHR workflow with a different version. Technology acceptance frameworks typically account for various variables to explain the intention and eventual use of a technological innovation. These variables encompass multiple concepts, such as individuals’ perceptions of usefulness, ease of use, and motivation. At present, it is unclear whether these variables also influence the intention to use and adopt a technological innovation in the same way for de-implementation contexts as they have for implementation contexts. For instance, clinicians’ motivation in de-implementation contexts may be affected by perceived harms to the patient or to themselves (e.g., litigation concerns) from continuing the use of a specific technology or its components. Consequently, similar to how other researchers have called for implementation science frameworks to be examined for their suitability in de-implementation research, there may also need to be similar efforts applied to examining technology acceptance frameworks in de-implementation contexts.

Third, although our findings are promising on leveraging the EHR to support de-implementation, additional research is needed to assess how the design of EHR-based intervention contributes to the fidelity of the de-implementation intervention. This will be especially important as different health systems use different EHR vendors. Differences in features and their sophistication across different EHR products may also exist. We saw that some studies examined the use of nudges, or interventions that involve changes to the interface that subtly guide end-users toward particular behaviors without forcing them towards specific actions. For instance, some studies examined how different nudging techniques, such as default dose amounts on order sets and suggesting alternatives at the point of care, can influence differences in clinicians’ EHR behaviors. Future research is needed to comparatively assess the effects of different nudging techniques on de-implementation behaviors. Another consideration is the level of detail presented within alerts that help justify the use of a suggested clinical intervention in response to a given diagnosis. Some studies included in this review reported the lack of or inadequate guidance on alternative treatments as a barrier to alert effectiveness. Future research is needed to better implement progressive disclosure principles (e.g., display all relevant information in alert vs. place rationales and additional information when clinician clicks on “Show More” or “Show All”) while balancing information overload and providing enough information at the point of care to assist in clinical decision-making.

Fourth, we attempted to map de-implementation outcomes collected across studies using Proctors et al.’s Implementation Outcomes framework. Although we found that all constructs (e.g., acceptability, sustainability) were overall represented, there were noticeable differences in the range of constructs examined when stratifying by type of EHR mechanism. For instance, we found that studies on EHR alerts examined all eight constructs while communication tools examined four. This may be, in part, due to the disproportionate number of studies on EHR alerts compared to communication tools. We also found that measurement approaches were highly variable. However, questions emerge on whether the approaches used were valid. To our knowledge, no validated de-implementation measure exists. Furthermore, no study used any of the many previously published and validated instruments assessing implementation measures when evaluating the de-implementation intervention [110–115]. Our findings suggest a persistent need for the development of validated de-implementation measures. This may require, in part, the adaptation and re-evaluation of existing implementation measures with psychometric evidence to see if they are as valid in de-implementation contexts.

Lastly, we identified a paucity of studies that reported on both the type and amount of costs involved in developing and implementing the de-implementation intervention. This finding was consistent with a past review of randomized studies of de-implementation interventions [116]. In contrast to implementing practices into care delivery, de-implementation may experience unique challenges if financial incentives are associated with a practice that is being targeted for de-implementation [117, 118]. Since leadership support is one of several key factors that have been identified as facilitators [119], they may require additional information on cost implications of de-implementing a practice before considering whether to endorse the de-implementation and commit resources. Accordingly, future research should report on the costs associated with developing and implementing the de-implementation intervention and any cost savings observed. This information may also be useful for organizations, such as safety net clinics, that need to prioritize initiatives based on impact on patient outcomes and costs. To assist with this, there may need to be guidance or standardization of what information to include when reporting on costs associated with de-implementation interventions. Some researchers have suggested itemization of costs involved, time range (e.g., $1,000 saved over a span of six months), and reporting separately the costs of developing the intervention vs. implementing it [116]. Some efforts to develop instruments for reporting costs are ongoing [120].

### Limitations and additional future research directions

In addition to the areas for future research discussed above, we offer several future research directions based on the limitations of this review. First, all included studies were targeting either ineffective care or inefficient care. To our knowledge, no study has attempted to target unwanted care (i.e., care that does not adhere to patients’ preferences). This pattern is consistent with the findings of a previous review [19], which raises a persistent need for research in this area. Second, our review identified four EHR intervention types to support de-implementation. There may be other EHR intervention types that may have potential to facilitate de-implementation, such as patient dashboards for flagging relevant patients in need of deprescribing interventions and patient portals for disseminating patient education materials. These represent additional areas for further research in addition to the lesser studied EHR intervention types discussed in this review. Third, in light of the relative absence of comparative evaluations, we are unable to draw definitive conclusions on the relative strength of the individual EHR types at facilitating de-implementation efforts (e.g., EHR alerts that advise against MRI orders for low back pain vs. order sets containing appropriate interventions). Consequently, future research might assess this to better clarify if any given intervention type has a larger effect size for specific circumstances. Lastly, most studies in this review used interventions that targeted physicians. Fewer studies targeted other members of the care team, such as physician assistants, nurse practitioners, and pharmacists. To our knowledge, no study targeted other roles, such as dietitians and psychologists. Since EHR workflows may vary by role, the findings reported in this review may not generalize to other members of the care team and additional studies are needed to examine whether an EHR de-implementation intervention’s effectiveness varies by type of care team member.

## Conclusion

EHR-based interventions can support de-implementation by better operationalizing clinical guidelines into changes in care. Our scoping review found that these interventions could be grouped into four key categories: EHR alerts, order sets and preference lists, documentation templates, and communication tools among care teams. While these interventions show promise, their effectiveness varied, with limited to modest evidence supporting their use. Our findings revealed that the evidence base is limited by the general lack of frameworks used, unclear theoretical rationale to support the use of selected de-implementation strategies, and the unclear validity of de-implementation outcomes used. Additional research is needed to develop and validate frameworks and outcomes for de-implementation to strengthen the evidence base.

## Supplementary Information

Additional file 1.

Additional file 2.

Additional file 3.

Additional file 4.

Additional file 5.

Additional file 6.

## Data Availability

All data used in this work have been presented.
