# Supplementary material for: The role of electronic health records systems in de-implementing low-value care in primary care: a scoping review
Source: Implement Sci Commun. 2025 Dec 19;6:138. doi: 10.1186/s43058-025-00826-6 (PMC12717702; doi:10.1186/s43058-025-00826-6)
Supplement: Supplementary file 2 — Additional file 2. [file 43058_2025_826_MOESM2_ESM.docx]

| Concept | MeSH terms | Search terms |
| --- | --- | --- |
| EHR | ("electronic health records"[MeSH Terms:NoExp]) OR ("medical record systems, computerized"[MeSH Terms]) OR ("clinical decision support system"[MeSH Terms]) OR ("medical order entry systems"[MeSH Terms]) OR ("electronic prescribing"[MeSH Terms]) | EHR OR EMR OR EHRs OR EMRs OR "electronic health record" OR "electronic health records" OR "electronic medical record" OR "electronic medical records" OR "computerized medical record" OR "computerized medical records" OR CDS OR “clinical decision support” OR CPOE OR “computerized physician order entry” OR “order entry” OR “computerized provider order entry” OR InBasket OR “In Basket” OR “patient messages” OR “patient-generated messages” OR “e-prescribing” OR “e-prescribe” OR “electronic prescription” OR “electronic prescriptions” OR “electronic prescribing” |
| De-implementation | ("implementation science"[MeSH Terms]) OR ("low-value care"[MeSH Terms]) | “De-implementation” OR “de-implementing” OR deimplement* OR “de-adoption” OR “de-adopting” OR deadopt* OR “de-prescribing” OR “de-prescribe” OR deprescri* OR discontinu* OR “de-listing” OR “de-list” OR delist* OR “dis-investment” OR “dis-investing” OR disinvest* OR “decrease use” OR “decreased use” OR “decreasing use” OR “de-commission” OR “de-commisioning” OR “de-commission” OR decommission* OR “de-funding” OR “de-fund” OR defund* OR “ex-novate” OR “ex-novation” OR exnovat* OR reassess* OR withdraw* OR contradict* OR refut* OR substitu* OR restrict* OR abandon* OR ceas* OR ending OR ended OR “choosing wisely” OR disadopt* OR “dis-adopting” OR “dis-adoption” OR divest* OR inapprop* OR ineffective* OR “low-value” OR “low value” OR obsolete OR outmode* OR overuse* OR reallocate* OR reassess* OR “re-assessing” OR “re-assessed” OR “medical revers*” OR supersed* OR unlearn* OR overtreat* OR overdiagnos* OR overmedicati* OR undiffus* OR stop* OR minimiz* OR relinquish* |
| Primary care | ("preventive medicine"[MeSH Terms]) OR ("early detection of cancer"[MeSH Terms]) OR ("vaccination"[MeSH Terms]) OR ("primary health care"[MeSH Terms]) | “Primary care” OR “preventive care” OR “preventative care” OR “preventive medicine” OR “family practic*” OR “general practic*” OR “primary healthcare” OR “primary health care” OR “preventive health” OR screening* OR vaccinat* OR immunization* OR “general health checkup*” OR “general health check-up*” OR “wellness visit*” OR “annual checkup*” OR “annual check-up*” OR “yearly checkup*” OR “yearly check-up*” OR “annual physical*” OR “yearly physical*” OR “general internal medicine” OR “general pediatric*” OR “family medicine” |
