# Supplementary material for: The role of electronic health records systems in de-implementing low-value care in primary care: a scoping review
Source: Implement Sci Commun. 2025 Dec 19;6:138. doi: 10.1186/s43058-025-00826-6 (PMC12717702; doi:10.1186/s43058-025-00826-6)
Supplement: Supplementary file 3 — Additional file 3. [file 43058_2025_826_MOESM3_ESM.pdf]

| Study | Aim of the Article | Country | Study Design | Type of Primary Care Setting | Medical Conditions Involved | Low-Value Care Targeted | Type of Low-Value Care | Type of Clinicians Targeted | Framework(s) Used | How EHR Was Involved | EHR Vendor | Sample Size | De-Implementation Outcomes Assessed | Intervention Effectiveness Outcomes | Intervention Effectiveness Comparison Group |
|---|---|---|---|---|---|---|---|---|---|---|---|---|---|---|---|
| Ackerman 2013 | - To assess de-implementation outcomes<br>- To identify barriers and facilitators | United States | Observational | Internal medicine, family medicine | Acute bronchitis | Antibiotic prescribing for acute bronchitis | Ineffective care | Physicians, NPs, PAs, nurses | PRECEDE-PROCEED | EHR alerts | Epic | 26 physicians, 2 NPs, 1 PA | Acceptability, adoption, appropriateness, feasibility | None | None |
| Alagiakrishnan 2016 | - To assess de-implementation outcomes<br>- To identify barriers and facilitators | Canada | Interviews | Geriatrics, family medicine | All | Inappropriate medication prescribing | Ineffective care | Physicians | Not reported | EHR alerts | Epic | 6 physicians, 2 geriatric medicine specialists | Acceptability, adoption, appropriateness, feasibility, fidelity | None | None |
| Alagiakrishnan 2019 | - To assess intervention effectiveness<br>- To identify barriers and facilitators | Canada | Quasi-experimental | Family practice, geriatrics | All | Inappropriate medication prescribing | Ineffective care | Physicians | Not reported | EHR alerts | Epic | 7,385 patients | None | De-prescribing rate | Pre-intervention |
| Ancker 2021 | - To assess intervention effectiveness<br>- To identify barriers and facilitators | United States | Quasi-experimental | Federally qualified health center (family medicine), vs. multispecialty practice | Chronic pain | Prescribing opioids for chronic pain | Ineffective care | Physicians, NPs | Not reported | Order sets and preference lists | Epic | 3,895 patients | None | Proportion of opioid prescriptions that were guideline-concordant | Pre-intervention |
| Anderson 2020 | - To assess intervention effectiveness<br>- To assess de-implementation outcomes<br>- To identify barriers and facilitators | United States | Quasi-experimental | Family medicine, internal medicine, geriatrics | All | Basic and comprehensive metabolic panel orders without clinical indications | Inefficient care | Physicians | Not reported | EHR alerts | Not reported | 240 physicians | Costs | Proportion of inappropriate blood chemistry panels | Pre-intervention |
| Buehrle 2020 | - To assess intervention effectiveness<br>- To identify barriers and facilitators | United States | Quasi-experimental | Not specified | All | Inappropriate antibiotic prescribing | Ineffective care | Not clear | Not reported | Order sets and preference lists | Not reported | Baseline Period: 28,402 office visits, Intervention Period: 32,982 office visits, Post-Intervention Period: 33,121 | None | Proportion of overall antibiotic prescribing, proportion of inappropriate antibiotic prescribing | Pre-intervention |
| Campbell 2021 | - To assess intervention effectiveness<br>- To assess de-implementation outcomes<br>- To identify barriers and facilitators | United States | RCT | Primary care, safety net | All | Prescribing high-risk anticholinergics | Ineffective care | Not reported | Not reported | EHR alerts | Epic | 552 patients | Fidelity | Proportion of discontinued orders | Standard of care |
| Cole 2020 | - To assess intervention effectiveness<br>- To identify barriers and facilitators | United Kingdom | Quasi-experimental | Not specified | Chronic obstructive pulmonary disease | Overuse of inhaled corticosteroids for chronic obstructive pulmonary disease | Ineffective care | Not reported | Not reported | EHR alerts | EMIS Web | 3,496 patients | None | Proportion of patients prescribed inhaled corticosteroids | Pre-intervention |
| Cossette 2019 | - To assess intervention effectiveness<br>- To assess de-implementation outcomes | Canada | Quasi-experimental | Family medicine, geriatrics | All | Medication issues | Ineffective care | Physicians | Knowledge-to-action | Communication tools within care team | Purkinje | 65 patients | Acceptability, appropriateness | Proportion of patients who experienced medication changes | Pre-intervention |

| | | | | | | | | | | | | | | | | |
|---|---|---|---|---|---|---|---|---|---|---|---|---|---|---|---|---|
| Delvaux 2020 | - To assess intervention effectiveness<br><br>- To identify barriers and facilitators | Belgium | RCT | General practice | Conditions requiring laboratory tests | Inappropriate laboratory test ordering | Inefficient care | Physicians | Not reported | EHR alerts | Not reported | 9,683 patients | None | Proportion of appropriate lab tests ordered, number of tests ordered | Standard of care |
| Feldstein 2006 | To assess intervention effectiveness | United States | Quasi-experimental | Unspecified | Not reported | Drug-drug interactions involving warfarin | Ineffective care | Physicians, NPs, PAs | Not reported | EHR alerts | Not reported | 4,743 patients | None | Number of overall warfarin-related interactions and number of warfarin-acetaminophen interactions | Pre-intervention |
| Fried 2017 | To assess intervention effectiveness | United States | RCT | Not reported | Hypertension and diabetes mellitus | Medication issues | Ineffective care | Not reported | Not reported | EHR alerts | Not reported | 128 patients | None | Proportion of medication reconciliation errors corrected | Standard of care |
| Gill 2011 | - To assess intervention effectiveness<br><br>- To assess de-implementation outcomes<br><br>- To identify barriers and facilitators | United States | RCT | Family medicine, general internal medicine, general practice | Peptic ulcers, gastritis and duodenitis, gastrointestinal hemorrhage | Inappropriate NSAID prescribing | Ineffective care | Physicians, NPs, PAs | Not reported | EHR alerts | Centricity Provider Office | 5,234 patients | Acceptability, adoption, feasibility, fidelity | Odds of guideline-concordant care provided, odds of prescribing new gastroprotective medications, odds of having NSAIDs discontinued | Standard of care |
| Gonzales 2013 | - To assess intervention effectiveness<br><br>- To assess de-implementation outcomes<br><br>- To identify barriers and facilitators | United States | RCT | Internal medicine, family medicine | Acute bronchitis | Antibiotic prescribing for acute bronchitis | Ineffective care | Physicians, NPs, PAs, nurses | PRECEDE-PROCEED | EHR alerts | Epic | 12,776 visits | Penetration | Odds of ordering antibiotic prescriptions | Standard of care |
| Gulliford 2019 | - To assess intervention effectiveness<br><br>- To assess de-implementation outcomes<br><br>- To identify barriers and facilitators | United Kingdom | RCT | General practice | Respiratory tract infections | Antibiotic prescribing for respiratory tract infections | Ineffective care | Physicians, NPs | Social cognitive theory | EHR alerts | Vision | 79 practices | Acceptability, adoption, costs, feasibility, fidelity, penetration | Relative risk of ordering antibiotic prescriptions | Standard of care |
| Hingorani 2015 | - To assess intervention effectiveness<br><br>- To assess de-implementation outcomes | United States | Quasi-experimental | Internal medicine | Upper respiratory tract infections | Antibiotic prescribing for upper respiratory tract infections | Ineffective care | Physicians, NPs | Not reported | EHR alerts | Epic | 240 patients | Penetration | Proportions of patients receiving guideline-adherent care | Pre-intervention |
| Howell 2014 | - To assess intervention effectiveness<br><br>- To identify barriers and facilitators | United States | Quasi-experimental | General internal medicine | All | Ordering Pap smears without clinical indications | Inefficient care | Physicians, nurses | Not reported | EHR alerts | Epic | 57,998 patients | None | Relative frequencies of Pap tests done | Pre-intervention |
| Ip 2014 | To assess intervention effectiveness | United States | Quasi-experimental | Unspecified | Low back pain | Lumbar MRI for low back pain | Inefficient care | Physicians | Not reported | EHR alerts | Not reported | 21,445 visits | None | Odds of ordering MRI orders, proportion of patients who had lumbar spine MRI orders within 30 days of a visit | Pre-intervention |

| | | | | | | | | | | | | | | | |
|---|---|---|---|---|---|---|---|---|---|---|---|---|---|---|---|
| Keohane 2017 | To assess intervention effectiveness | United Kingdom | Quasi-experimental | Not reported | Chronic kidney disease | Prescribing NSAIDs for chronic kidney disease | Ineffective care | Not reported | Not reported | EHR alerts | HealthOne | Unclear | None | Proportion of NSAIDs prescribed | Pre-intervention |
| Khadadah 2022 | - To assess intervention effectiveness<br><br>- To assess de-implementation outcomes | Canada | Quasi-experimental | Family medicine | Bleeding disorders | Coagulation lab testing | Ineffective care | Physicians, NPs | Not reported | Order sets and preference lists | Not reported | Not reported | Costs | Rate of activated partial thromboplastin time testing, rate of prothrombin time/international normalized ratio | Pre-intervention |
| Lagisetty 2020 | - To assess intervention effectiveness<br><br>- To assess de-implementation outcomes<br><br>- To identify barriers and facilitators | United States | Quasi-experimental | Family medicine, internal medicine | Chronic pain | Prescribing opioids for chronic pain | Ineffective care | Physicians | Not reported | Communication tools within care team | Not reported | 46 patients | Acceptability, appropriateness, feasibility, sustainability | Average oral morphine equivalents | Pre-intervention |
| Liebschutz 2017 | - To assess intervention effectiveness<br><br>- To assess de-implementation outcomes<br><br>- To identify barriers and facilitators | United States | RCT | Safety net (internal medicine, family medicine) | Pain management | Prescribing opioids for pain management | Ineffective care | Physicians, NPs | Not reported | Communication tools within care team | Not reported | 985 patients | Sustainability | Odds of discontinuing opioid treatments, odds of reducing opioid dose amounts by at least 10% | Standard of care |
| Lin 2020 | - To assess intervention effectiveness<br><br>- To identify barriers and facilitators | United States | Quasi-experimental | Not specified | Cystitis, bronchitis, sinusitis | Fluoroquinolone prescribing for cystistis, bronchitis, and sinusitis | Ineffective care | Physicians, APPs | Not reported | Order sets and preference lists | Epic | 1,033 prescriptions | None | Proportion of fluroquinolone prescriptions that were inappropriate | Pre-intervention |
| Litvin 2012 | - To assess de-implementation outcomes<br><br>- To identify barriers and facilitators | United States | Quasi-experimental | Unspecified | Respiratory tract infections | Prescribing antbiotics for viral respiratory tract infections | Ineffective care | Physicians, NPs, PAs | Not reported | Documentation templates | McKesson Practice Partner | 27 physicians, 6 NPs, 6 PAs | Adoption, appropriateness, feasibility, fidelity, penetration | None | None |
| Litvin 2013 | - To assess intervention effectiveness<br><br>- To assess de-implementation outcomes<br><br>- To identify barriers and facilitators | United States | Quasi-experimental | Unspecified | Respiratory tract infections | Prescribing antbiotics for viral respiratory tract infections | Ineffective care | Physicians, NPs, PAs | Not reported | Documentation templates | McKesson Practice Partner | 38,592 visits | Appropriateness, feasibility | Proportion of patients on inappropriate antibiotic use, proportion of patients using broad spectrum antibiotics | Pre-intervention |
| Mainous 2013 | - To assess intervention effectiveness<br><br>- To assess de-implementation outcomes<br><br>- To identify barriers and facilitators | United States | Quasi-experimental | Unspecified | Respiratory tract infections | Prescribing antbiotics for viral respiratory tract infections | Ineffective care | Physicians, NPs, PAs | Not reported | Documentation templates | McKesson Practice Partner | 70 practices | Adoption, penetration | Proportion of patients on inappropriate antibiotic use, proportion of patients using broad spectrum antibiotics | Pre-intervention |

| | | | | | | | | | | | | | | | |
|---|---|---|---|---|---|---|---|---|---|---|---|---|---|---|---|
| Mann 2020 | - To assess intervention effectiveness<br><br>- To assess de-implementation outcomes<br><br>- To identify barriers and facilitators | United States | RCT | Internal medicine, family medicine | Acute respiratory tract infections | Antibiotic prescribing | Ineffective care | Physicians, NPs, PAs | RE-AIM | EHR alerts, order sets and preference lists | Not reported | 100,573 patients | Adoption, fidelity, penetration, sustainability | Relative risk of antibiotic prescriptions, relative risk of inappropriate antibiotic prescribing | Standard of care |
| Martins 2017 | To assess intervention effectiveness | Portugal | RCT | Family medicine | All | Ordering labs without clinical indications | Inefficient care | Physicians | Not reported | Order sets and preference lists | Sistema de Apoio ao Médico | 190,633 patients | None | Number of unnecessary tests | Standard of care |
| Matulis 2017 | - To assess intervention effectiveness<br><br>- To identify barriers and facilitators | United States | Quasi-experimental | Internal medicine | Patients undergoing low-risk surgery | Unnecessary preoperative testing | Inefficient care | Not reported | Not reported | Order sets and preference lists | Epic | 547 visits | None | Proportion of patients receiving unnecessary tests | Pre-intervention |
| McCormick 2020 | To assess intervention effectiveness | United States | Quasi-experimental | Family medicine | Uncomplicated cystitis, pyelonephritis | Antibiotic prescribing for urinary tract infections | Ineffective care | Physicians | Not reported | Documentation templates, order sets | Not reported | 162 patients | None | Odds of having an appropriate antibiotic, dose, and duration | Pre-intervention |
| McDermott 2014 | - To assess de-implementation outcomes<br><br>- To identify barriers and facilitators | United Kingdom | Observational | General practice | Acute respiratory infections | Antibiotics prescribing for respiratory tract infections | Ineffective care | Not reported | Linnan and Steckler's framework for evaluating public health interventions | EHR alerts | Not reported | 103 Clinicians | Acceptability, appropriateness, feasibility, fidelity | None | None |
| Meeker 2016 | To assess de-implementation outcomes | United States | RCT | Internal medicine, family medicine | Respiratory tract infections | Prescribing antibiotics for viral respiratory tract infections | Ineffective care | Physicians, NPs, PAs | Not reported | EHR alerts | Not reported | 47 practices | None | Proportion of patients receiving antibiotics | Standard of care |
| Milone 2014 | To assess intervention effectiveness | United States | Descriptive | Family medicine, safety net | All | Medication issues | Ineffective care | Physicians | Not reported | Communication tools within care team | Not reported | 327 patients | None | Proportion of patients who had discrepancies identified in medication lists | None |
| Nallapeta 2020 | - To assess intervention effectiveness<br><br>- To identify barriers and facilitators | United States | Quasi-experimental | Internal medicine, safety net | gastroesophageal refulx disease | Prescribing proton pump inhibitors for gastroesophageal reflux disease | Inefficient care | Physicians | Not reported | Documentation templates | Not reported | 180 patients | None | Proportion of patients who had proton pump inhibitors discontinued | Pre-intervention |
| Odenthal 2020 | - To assess intervention effectiveness<br><br>- To identify barriers and facilitators | United States | Descriptive | Family medicine, safety net | GERD w/o esophagitis or w/o clear indication | PPI deprescribing | Inefficient care | Pharmacists | Not reported | Communication tools within care team | Epic | 22 patients | None | Proportion of patients whose proton pump inhibitors were discontinued | None |
| Persell 2016 | - To assess intervention effectiveness<br><br>- To assess de-implementation outcomes | United States | Quasi-experimental | General interal medicine | Acute respiratory infections | Antibiotic prescribing for acute respiratory infections (ARIs) | Ineffective care | Physicians, NPs | Not reported | EHR alerts | Epic | 27 internists and 1 nurse practitioner | Adoption | Odds of prescribing antibiotics for inappropriate diagnoses | Pre-intervention |
| Petrilli 2018 | To assess intervention effectiveness | United States | Quasi-experimental | Internal medicine, family medicine, general pediatrics, geriatrics | All | Vitamin D lab ordering | Inefficient care | Not reported | Not reported | EHR alerts | Not reported | 32,035 orders | None | Relative risk of vitamin D testing | Pre-intervention |

| | | | | | | | | | | | | | | | |
|---|---|---|---|---|---|---|---|---|---|---|---|---|---|---|---|
| Rowe 2023 | - To assess intervention effectiveness<br><br>- To assess de-implementation outcomes | United States | Quasi-experimental | Internal medicine, geriatrics | Cancer and urine screening, diabetes | Overordering of prostate-specific antigen testing among older men, urinalysis and urine culture for non-specific reasons in older women, use of insulin or oral hypoglycemic medications in older adults | Inefficient care | Physicians | Not reported | EHR alerts | Not reported | 14 PCP | Acceptability, appropriateness, feasibility, fidelity | Differences in differences in prostate-specific antigen tsting, urinalysis/culture orders, or diabetes overtreatment | Non-exposed physicians |
| Rozario 2020 | - To assess intervention effectiveness<br><br>- To assess de-implementation outcomes<br><br>- To identify barriers and facilitators | United States | Quasi-experimental | Family medicine, internal medicine | All | Vitamin D screening | Inefficient care | Physicians, NPs, PAs | Not reported | Order sets and preference lists | Cerner | 587,506 visits | Costs | Rates of vitamin D orders placed | Pre-intervention |
| Seppänen 2016 | - To assess intervention effectiveness<br><br>- To identify barriers and facilitators | Finland | Quasi-experimental | Not specified | All | Ordering erythrocyte sedimentation rate and aspartate transaminase tests | Inefficient care | Physicians | Not reported | Order sets and preference lists | Not reported | 272 physicians | None | Risk ratios of ordering aspartate transaminase and erythrocyte sedimentation rate tests | Pre-intervention |
| Shelton 2015 | - To assess intervention effectiveness<br><br>- To identify barriers and facilitators | United States | Quasi-experimental | Not specified | Cancer screening | Prostate-specific antigen testing among older men | Inefficient care | Not reported | Not reported | EHR alerts | Not reported | 30,150 patients | None | Rate ratios of prostate-specific antigen testing | Pre-intervention |
| Singh-Franco 2022 | - To assess intervention effectiveness<br><br>- To identify barriers and facilitators | United States | Descriptive | Not reported | Upper respiratory infections | Antibiotic prescribing for upper respiratory infections | Ineffective care | Physicians, NPs, PAs | Not reported | Order sets and preference lists, communication tools within care team | Epic | 2,934 antibiotic orders | None | Proportion of antibiotic orders deemed unnecessary, proportion of necessary antibiotic orders deemed inappropriate | None |
| Singhal 2022 | - To assess intervention effectiveness<br><br>- To identify barriers and facilitators | United States | Descriptive | Not specified | All among older adults | Prescribing benzodiazepines and sedative-hypnotics | Ineffective care | Not reported | Not reported | EHR alerts | Not reported | 8,667 alert triggers | None | Proportion of clinicians following alert guidance | None |
| Tamblyn 2003 | - To assess intervention effectiveness<br><br>- To assess de-implementation outcomes<br><br>- To identify barriers and facilitators | Canada | RCT | Not specified | All | Inappropriate prescribing | Ineffective care | Physicians | Not reported | EHR alerts | Not reported | 12,560 patients | Adoption | Relative risk of inappropriate prescribing | Standard of care |
| Tamblyn 2008 | - To assess intervention effectiveness<br><br>- To identify barriers and facilitators | Canada | RCT | General practice, family medicine | All | Medication issues | Ineffective care | Physicians | Not reported | EHR alerts | Not reported | 3,449 patients | None | Odds of therapeutic duplication, odds of contraindications | On-demand decision support |
| vanWijk 2001 | To assess intervention effectiveness | The Netherlands | RCT | General practice | All | Inappropriate lab orders placed | Inefficient care | Physicians | Not reported | Order sets and preference lists | Not reported | 7,094 patients | None | Relative risk of ordering tests | Clinical guidelines |

| | | | | | | | | | | | | | | | |
|---|---|---|---|---|---|---|---|---|---|---|---|---|---|---|---|
| Vardy 2005 | To assess intervention effectiveness | Israel | Quasi-experimental | Family medicine | All | Inappropriate lab orders placed | Inefficient care | Physicians | Not reported | Order sets and preference lists | Not reported | Not reported | None | Total number of tests | Pre-intervention |
| Walsh 2016 | - To assess intervention effectiveness<br><br>- To assess de-implementation outcomes<br><br>- To identify barriers and facilitators | Canada | Quasi-experimental | Family medicine | Gastroesophageal reflux disease, endoscopic negative reflux disease | Taking proton pump inhibitors without clinical indications | Inefficient care | Physicians, NPs | Not reported | EHR alerts | Not reported | 46 patients | Acceptability, feasibility, penetration | Proportion of patients deprescribed proton pump inhibitors | Pre-intervention |
| Wessell 2013 | To assess intervention effectiveness | United States | Quasi-experimental | Family medicine, internal medicine | All | Medication issues | Ineffective care | Not reported | Authors developed medication safety framework for primary care officebased practices | EHR alerts | McKesson Practice Partner | 20 practices | None | Proportion of patients who avoided potentially inappropriate therapy, proportion of patients who avoided potential drug-disease interactions, proportion of patients who avoided potentially inappropriate dosing, proportion of patients who avoided potential drug-drug interactions | Pre-intervention |
| Wong 2019 | - To assess intervention effectiveness<br><br>- To identify barriers and facilitators | United States | Quasi-experimental | Internal Medicine | Chronic pain | Opioid prescribing for chronic pain | Ineffective care | Physicians | Not reported | Documentation templates | Cerner | 58 patients | None | Average daily morphine milligram equivalents | Pre-intervention |
