# Supplementary material for: The role of electronic health records systems in de-implementing low-value care in primary care: a scoping review
Source: Implement Sci Commun. 2025 Dec 19;6:138. doi: 10.1186/s43058-025-00826-6 (PMC12717702; doi:10.1186/s43058-025-00826-6)
Supplement: Supplementary file 4 — Additional file 4. [file 43058_2025_826_MOESM4_ESM.docx]

| EHR Intervention Type | Type of Finding | Specific Findings | Citation |
| --- | --- | --- | --- |
| EHR Alerts | Favorable | Reduced the proportion of patients with inappropriate lab orders (percent difference = -13.0%, 95% CI -15.3 - -10.6) | Anderson et al., 2020 |
|  |  | Reduced the proportion of patients who were prescribed inhaled corticosteroids (percent difference = -2.1%, p=0.02) | Cole et al., 2020 |
|  |  | There was an increase in the proportion of appropriate tests ordered (percent difference = -21%, 95% CI 16% - 26%) and a decrease in the number of tests ordered (mean difference = -7.15, 95% CI 3.37 - 10.93) | Delvaux et al., 2020 |
|  |  | Reduced the number of overall warfarin-related interactions (-489.8, 95% CI -664.9 - -314.7) and warfarin-acetaminophen interactions (-503.4, 95% CI -670.0 - -336.8) | Feldstein et al., 2006 |
|  |  | Compared to control, there was an increase in the proportion of medication reconciliation errors corrected (14.3% vs. 48.4%, p<0.001) | Fried et al., 2017 |
|  |  | Lower odds of antibiotic prescriptions made (OR = 0.64, 95% CI 0.45 - 0.91) | Gonzales et al., 2013 |
|  |  | Lower relative risk of antibiotic prescriptions made (RR = 0.88, 95% CI 0.78 - 0.99) | Gulliford et al., 2019 |
|  |  | Compared to baseline, there were higher proportions of patients receiving guideline-adherent care for patients with sinusitis (57.6% vs. 90.9%, p<0.001), pharyngitis (25.0% vs. 64.3%, p=0.003), upper respiratory infections (88.6% vs. 96.2%, p=0.008), and acute respiratory infections (78.7% vs. 91.3%, p<0.001). | Hingorani et al., 2015 |
|  |  | There were lower relative frequencies of Pap tests done for patients under 21 years old (0.28, 95% CI 0.21 - 0.36) and over 71 years old (0.69, 95% CI 0.57 - 0.83) | Howell et al., 2014 |
|  |  | There were lower odds of patients receiving MRI orders (OR = 0.68, 95% CI 0.59 - 0.77). Compared to baseline, there was also a reduction in the proportion of patients who had lumbar spine MRI orders placed within 30 days of a visit (6.7% vs. 5.1%, p<0.001). | Ip et al., 2014 |
|  |  | There was lower relative risk of vitamin D testing (RR = 0.89, 95% CI 0.83 - 0.96) | Petrilli et al., 2018 |
|  |  | There were lower rate ratios of prostate-specific antigen screening when the alerts were first used (0.78, 95% CI 0.72 - 0.85). There were higher rate ratios when the alert was initially turned off (1.16, 95% CI 1.07 - 1.26). There were lower rate ratios again when the alert was turned back on (0.90, 0.83 - 0.97). There were higher rate ratios again when the alert was turned back off (1.16, 1.07 - 1.27). | Shelton et al., 2015 |
|  | Mixed | There were higher odds of guideline-concordant care provided (OR = 1.19, 95% CI 1.01 - 1.42) and higher odds of prescribing new gastroprotective medications (OR = 1.33, 95% CI 1.01 - 1.74).  No differences in the odds of having NSAIDs discontinued occurred (OR = 1.18, 95% CI 0.99 - 1.40) | Gill et al., 2011 |
|  |  | There were a lower proportion of patients receiving antibiotics when the alert incorporated accountable justification nudging techniques (percent difference = -7%, 95% CI -9.1 - -2.9) but not for suggested alternatives nudging techniques (percent difference = -5%, 95% CI -7.8 - 0.1) | Meeker et al., 2016 |
|  |  | There were lower relative risk of inappropriate prescribing that led to drug-age contraindications (RR = 0.77, 95% CI 0.59 - 1.00) and excessive duration of therapy (RR = 0.78, 95% CI 0.61 - 0.99),  There were no differences in relative risk of inappropriate prescribing that led to drug-disease contraindications (RR = 0.89, 95% CI 0.72 - 1.10), therapeutic duplication (RR = 0.87, 95% CI 0.69 - 1.11), or drug interactions (RR = 1.12, 95% CI 0.68 - 1.87). Similarly, there were no differences in relative risk of inappropriate discontinuation that led to drug-disease contraindications (RR = 1.08, 95% CI 0.85 - 1.36), drug-age contraindications (RR = 0.94, 0.79 - 1.13), excessive duration of therapy (RR = 1.00, 95% CI 0.77 - 1.29), therapeutic duplication (RR = 0.94, 95% CI 0.59 - 1.51), or drug interactions (RR = 1.33, 95% CI 0.90 - 1.95). | Tamblyn et al., 2003 |
|  |  | There were lower odds of therapeutic duplication among medications (OR = 0.43, 95% CI 0.29 - 0.64).  There were no differences in the odds of drug-disease contraindications (OR = 1.09, 95% CI 0.83 - 1.42), cumulative toxicity (OR = 1.71, 95% CI 0.77 - 3.79), drug interactions (0.91, 95% CI 0.79 - 2.52), drug-age contraindications (1.41, 95% CI 0.79 - 2.52), and dosing errors (OR = 1.10, 95% CI 0.55 - 2.19). | Tamblyn et al., 2008 |
|  |  | There were higher proportions of patients who avoided potentially inappropriate therapy (4.0%, 95% CI 1.5 - 11.3) and avoided potential drug-disease interactions (2.4%, 95% CI 0.5 - 4.2).  There were no differences in the proportion of patients who avoided potentially inappropriate dosing (2.6%, 95% CI -3.3 - 8.1) or avoided potential drug-drug interactions (-0.2%, 95% CI -0.5 - 0.4). | Wessell et al., 2013 |
|  | Null | Did not increase de-prescribing rate (proportion difference: 1.20%, p>0.05) | Alagiakrishnan et al., 2019 |
|  |  | Compared to baseline, there was no improvement in the percentage of discontinued orders (7.3% vs. 7.8%, p=0.65) | Campbell et al., 2021 |
|  |  | No differences in relative risk of either overall antibiotic prescription rates (RR = 0.99, 95% CI 0.87 - 1.11) or total inapproproate antibiotic prescribing (RR = 0.90, 95% CI 0.70 - 1.20) | Mann et al., 2020 |
|  |  | The odds of prescribing antibiotics for inappropriate diagnoses did not change regardless of using accountable justifications (OR = 0.98, 95% CI 0.42 - 2.29) or suggested alternatives nudging techniques (OR = 0.68, 95% CI (0.29 - 1.58) | Persell et al., 2016 |
|  |  | There were no differences in prostate-specific antigen testing (DiD = 0.03, 95% CI -5.6 - 5.7), urinalysis/culture orders (DiD = 1.8, 95% CI -5.4 - 9.0), or diabetes overtreatment (DiD = -4.3 - 12.6) | Rowe et al., 2023 |
|  | Unfavorable | No findings | --- |
|  | No Significance Testing or Quantitative Results | Reported only facilitators and barriers | Ackerman et al., 2013 |
|  |  | Reported only facilitators and barriers | Alagiakrishnan et al., 2016 |
|  |  | There was a 75% reduction in NSAIDs prescribed and a 90% reduction in repeat prescriptions of NSAIDs. No variance estimates were reported and no significance testing was done. | Keohane et al., 2017 |
|  |  | Reported only facilitators and barriers | McDermott et al., 2014 |
|  |  | 0.74% of clinicians were following alert guidance. No variance estimates were reported and no significance testing was done. | Singhal et al., 2022 |
|  |  | 26% of patients were deprescribed proton pump inhibitors. No variance estimates were reported and no significance testing was done. | Walsh et al., 2016 |
| Order Sets and Preference Lists | Favorable | Reduced the proportion of overall antibiotic prescribing (proportion difference = -26.8%, p<0.001) and inappropriate antibiotic prescribing (proportion difference = -24.7%, p=0.01) | Buehrle et al., 2020 |
|  |  | Compared to usual care, there was a lower proportion of patients receiving unnecessary tests (23% vs. 4%, p<0.001) | Matulis et al., 2017 |
|  |  | Improved odds of having an appropriate antibiotic, dose, and duration simultaneously (OR=4.5, 95% CI 2.3 - 9.0). | McCormick et al., 2020 |
|  |  | Reduced rates of vitamin D orders placed (-1.74%, -2.31 - -1.16) | Rozario et al., 2020 |
|  |  | Lower risk ratios of ordering aspartate transaminase tests (0.32, 95% CI 0.31 - 0.33) and erythrocyte sedimentation rate tests (0.44, 95% CI 0.42 - 0.45) | Seppänen et al., 2016 |
|  |  | Compared to an order set design that displayed possible orders based on clinical indication entered, the relative risk ratios of ordering tests was higher for the order set design that initially displayed a universally restricted number of orders (1.19, 95% CI 1.10 - 1.29). | van Wijk et al., 2001 |
|  | Mixed | Compared to baseline, the rate of activated partial thromboplastin time testing decreased-for the blood assessment tool only (rate difference = -0.14, p=0.001) but the rate was not reduced for the blood assessment tool and order set combined (rate difference = -0.12, p=0.25).  Compared to baseline, the rate of prothrombin time/international normalized ratio did not change for the blood assessment tool only (rate difference = -0.04, p=0.20) but did decrease for the blood assessment tool and order set combined (rate difference = -0.23, p<0.001). | Khadadah et al., 2022 |
|  |  | Compared to baseline, there were reductions in the number of unnecessary uric acid tests (10.5 vs. 4.6, p<0.001), serum protein electrophoresis tests (3.2 vs. 0.6, p<0.001), sedimentation rate tests (7.5 vs. 2.0, p<0.001), cancer antigen 19-9 tests (0.17 vs. 0.08, p<0.001), exercise electrocardiograms (0.56 vs. 0.38, p=0.028), carotid artery ultrasounds (0.25 vs. 0.15, p=0.021), Hepatitis B surface antigen tests (1.59 vs. 1.24, p=0.028), and Hepatitis C antibody tests (1.18 vs. 0.91, p=0.014).  Compared to baseline, no reductions occurred in the number of unnecessary rest electrocardiography tests (6.4 vs. 5.4, p=0.070), lung x-rays (1.9 vs. 1.4, p=1.000), pelvic ultrasounds (1.04 vs. 1.08, p=1.000), or spirometries (0.34 vs. 0.52, p = 0.812). | Martins et al., 2017 |
|  | Null | Proportion difference of guideline-concordant opioid prescriptions was 7% (p>0.05) | Ancker et al., 2021 |
|  |  | Compared to baseline, there was no difference in proportion of inappropriate fluoroquinolone prescriptions (74% vs. 64%, p=0.22) | Lin et al., 2020 |
|  |  | No differences in relative risk of either overall antibiotic prescription rates (RR = 0.99, 95% CI 0.87 - 1.11) or total inapproproate antibiotic prescribing (RR = 0.90, 95% CI 0.70 - 1.20) | Mann et al., 2020 |
|  | Unfavorable | No findings | --- |
|  | No Significance Testing or Quantitative Results | The proportion of patients whose antibiotics were deemed unnecessary was 12.6%. The proportion of necessary antibiotic orders that were inappropriate (e.g., excessive dose or treatment length) was 43.5%. No variance estimates were reported and no comparison groups were used. | Singh-Franco et al., 2022 |
|  |  | There was a 2% reduction in the total number of tests and a 4% reduction in number of tests per member-age. No significance testing was done. | Vardy et al., 2005 |
| Documentation Templates | Favorable | Improved odds of having an appropriate antibiotic, dose, and duration simultaneously (OR=4.5, 95% CI 2.3 - 9.0). | McCormick et al., 2020 |
|  |  | Compared to baseline, there was higher proportions of patients who had proton pump inhibitors discontinued (2.0% vs. 32.0%, p<0.05). | Nallapeta et al., 2020 |
|  |  | Compared to baseline, there was lower average daily morphine milligram equivalents (96.6 v. 67.6, p<0.001). | Wong et al., 2019 |
|  | Mixed | No differences in percent change of patients on inappropriate antibiotic use among both adults (1.57% [95% CI -5.35 - 8.49]) and children ([-1.89 [95% CI -9.03 - 5.26]).  However, there were reduced proportions of patients using broad spectrum antibiotics among both adults (-16.30% [95% CI -24.81 - -7.79]) and children (-16.30% [95% CI -23.29 - -9.31]). | Litvin et al., 2013 |
|  |  | Compared to control patients, there were reduced proportions of adults with inappropriate antibiotic use (4.2% vs. 0.6%, p=0.03). This was not observed among children (4.2% vs. 1.4%, p=0.34).  Compared to control patients, there were reduced proportions of patients using broad spectrum antibiotics among both adults (1.2% vs. -16.6%, p<0.001) and children (0.9% vs. -19.7%, p<0.001). | Mainous et al., 2013 |
|  | Null | No findings | --- |
|  | Unfavorable | No findings | --- |
|  | No Significance Testing or Quantitative Results | Reported only facilitators and barriers | Litvin et al., 2012 |
| Communication Tools Among the Care Team | Favorable | Higher odds of discontinuing opioid treatments (OR = 1.4, 95% CI 1.02 - 2.10) and reducing opioid dose amounts by at least 10% (OR = 1.6, 95% CI 1.10 - 2.40) | Liebschutz et al., 2017 |
|  | Mixed | No findings | --- |
|  | Null | No difference in average oral morphine equivalents prescribed at baseline and post-implementation (36.0 [SD: 6.8] vs. 29.1 [5.6], p=0.23) | Lagisetty et al., 2020 |
|  | Unfavorable | No findings | --- |
|  | No Significance Testing or Quantitative Results | The proportion of patients who experienced medication changes was 63.0% (95% CI 44.7 - 84.2). No comparison group was used. | Cossette et al., 2019 |
|  |  | The proportion of patients who had discrepancies identified in medication lists was 98.5%. No variance estimate was reported and no comparison group was used. | Milone et al., 2014 |
|  |  | The proportion of patients who was discontinued off proton pump inhibitors was 86%. Another 9% of patients instead had doses decreased. Another 5% could neither be discontinued nor have doses reduced. No variance estimates were reported and no comparison group was used. | Odenthal et al., 2020 |
|  |  | The proportion of patients whose antibiotics were deemed unnecessary was 12.6%. The proportion of necessary antibiotic orders that were inappropriate (e.g., excessive dose or treatment length) was 43.5%. No variance estimates were reported and no comparison groups were used. | Singh-Franco et al., 2022 |
| a. Abbreviations included odds ratios (OR), confidence intervals (CI), relative risk (RR), non-steroidal anti-inflammatory drugs (NSAIDs), and difference-in-differences (DiD). | | | |
