# Supplementary material for: The role of electronic health records systems in de-implementing low-value care in primary care: a scoping review
Source: Implement Sci Commun. 2025 Dec 19;6:138. doi: 10.1186/s43058-025-00826-6 (PMC12717702; doi:10.1186/s43058-025-00826-6)
Supplement: Supplementary file 5 — Additional file 5. [file 43058_2025_826_MOESM5_ESM.docx]

| **EHR Intervention Type** | **Study** | **Use Evaluative and Iterative Strategies** | **Provide Interactive Assistance** | **Adapt and Tailor to the Context** | **Develop Stakeholder Interrelationships** | **Train and Educate Stakeholders** | **Support Clinicians** | **Engage Consumers** | **Utilize Financial Strategies** | **Change infrastructure** | **Total** |
| --- | --- | --- | --- | --- | --- | --- | --- | --- | --- | --- | --- |
| Communication tools within care team | Cossette 2019 |  |  | Tailor strategies | Use advisory boards and workgroups | Conduct educational meetings, Distribute educational materials | Revise professional roles |  |  | Change record systems | 6 |
| Communication tools within care team | Lagisetty 2020 |  |  |  |  |  | Revise professional roles |  |  | Change record systems | 2 |
| Communication tools within care team | Liebschutz 2017 | Stage implementation |  |  | Identify and prepare champions | Conduct educational meetings | Revise professional roles |  |  | Change record systems | 5 |
| Communication tools within care team | Milone 2014 |  |  |  |  |  | Revise professional roles |  |  |  | 1 |
| Communication tools within care team | Odenthal 2020 |  |  |  |  | Distribute educational materials | Revise professional roles |  |  | Change record systems | 3 |
| Communication tools within care team | Singh-Franco 2022 |  |  |  |  | Distribute educational materials | Remind clinicians | Involve patients/consumers and family members |  | Change record systems | 4 |
| Documentation templates | Litvin 2012 | Audit and provide feedback |  |  | Identify and prepare champions, Organize clinician implementation team meetings | Conduct educational meetings; Conduct educational outreach visits | Remind clinicians |  |  | Change record systems | 7 |
| Documentation templates | Litvin 2013 | Audit and provide feedback |  |  | Identify and prepare champions | Conduct educational meetings; Conduct educational outreach visits | Remind clinicians |  |  | Change record systems | 6 |
| Documentation templates | Mainous 2013 | Audit and provide feedback |  |  | Identify and prepare champions | Conduct educational meetings; Conduct educational outreach visits | Remind clinicians |  |  | Change record systems | 6 |
| Documentation templates | Nallapeta 2020 | Assess for readiness and identify barriers and facilitators |  |  |  | Conduct educational meetings, Develop educational materials, Distribute educational materials | Remind clinicians | Involve patients/consumers and family members |  | Change record systems | 7 |
| Documentation templates | Wong 2019 |  |  |  |  | Conduct educational meetings | Remind clinicians |  |  | Change record systems, Mandate change | 4 |
| Documentation templates | McCormick 2020 | Conduct local needs assessment |  |  |  | Conduct educational meetings | Remind clinicians |  |  | Change record systems | 4 |
| EHR alerts | Ackerman 2013 |  |  |  | Identify and prepare champions, Organize clinician implementation team meetings | Conduct educational meetings, Distribute educational materials, Use train-the-trainer strategies | Remind clinicians | Involve patients/consumers and family members |  | Change record systems | 8 |
| EHR alerts | Alagiakrishnan 2016 |  |  |  |  |  | Remind clinicians |  |  | Change record systems | 2 |
| EHR alerts | Alagiakrishnan 2019 |  |  |  |  |  | Remind clinicians |  |  | Change record systems | 2 |
| EHR alerts | Anderson 2020 | Conduct local needs assessment |  |  | Use advisory boards and workgroups |  | Remind clinicians |  | Alter patient/consumer fees | Change record systems | 5 |
| EHR alerts | Campbell 2021 | Conduct local needs assessment, Stage implementation |  |  | Involve executive boards, Use advisory boards and workgroups | Develop educational materials, Distribute educational materials | Remind clinicians | Involve patients/consumers and family members |  | Change record systems | 9 |
| EHR alerts | Cole 2020 | Audit and provide feedback |  |  | Use advisory boards and workgroups | Conduct educational meetings, Develop educational materials, Distribute educational materials | Remind clinicians |  | Alter incentive/allowance structures | Change record systems | 8 |
| EHR alerts | Delvaux 2020 |  |  | Tailor strategies |  | Conduct educational meetings | Remind clinicians |  |  | Change record systems | 4 |
| EHR alerts | Feldstein 2006 | Assess for readiness and identify barriers and facilitators |  |  |  | Conduct educational meetings, Distribute educational materials | Remind clinicians |  |  | Change record systems | 5 |
| EHR alerts | Fried 2017 |  |  |  |  |  | Facilitate relay of clinical data to providers, Remind clinicians | Involve patients/consumers and family members |  | Change record systems | 4 |
| EHR alerts | Gill 2011 | Stage implementation |  |  | Use advisory boards and workgroups | Conduct educational meetings, Distribute educational materials | Remind clinicians |  |  | Change record systems | 6 |
| EHR alerts | Gonzales 2013 | Audit and provide feedback |  |  | Identify and prepare champions | Conduct educational meetings, Distribute educational materials | Remind clinicians | Involve patients/consumers and family members |  | Change record systems | 7 |
| EHR alerts | Gulliford 2019 | Audit and provide feedback; Obtain and use patients/consumers and family feedback |  |  | Use advisory boards and workgroups | Develop educational materials, Distribute educational materials | Remind clinicians | Intervene with patients/consumers to enhance uptake and adherence, Involve patients/consumers and family members |  | Change record systems | 9 |
| EHR alerts | Hingorani 2015 | Audit and provide feedback, Conduct local needs assessment |  |  | Develop academic partnerships | Conduct educational meetings, Conduct ongoing training, Work with educational institutions | Remind clinicians | Involve patients/consumers and family members |  | Change record systems | 9 |
| EHR alerts | Howell 2014 |  | Centralize technical assistance |  | Identify and prepare champions |  | Remind clinicians |  |  | Change record systems | 4 |
| EHR alerts | Ip 2014 | Audit and provide feedback |  |  |  |  | Remind clinicians |  |  | Change record systems | 3 |
| EHR alerts | Keohane 2017 | Audit and provide feedback |  |  |  | Distribute educational materials | Remind clinicians |  |  | Change record systems | 4 |
| EHR alerts | McDermott 2014 |  |  |  |  | Conduct educational meetings | Remind clinicians |  |  | Change record systems | 3 |
| EHR alerts | Meeker 2016 | Audit and provide feedback |  |  |  |  | Remind clinicians |  | Alter incentive/allowance structures | Change record systems | 4 |
| EHR alerts | Persell 2016 | Audit and provide feedback, Stage implementation |  |  |  |  | Remind clinicians |  | Alter incentive/allowance structures | Change record systems | 5 |
| EHR alerts | Petrilli 2018 |  |  |  | Involve executive boards, Use advisory boards and workgroups | Develop educational materials, Distribute educational materials |  |  |  |  | 4 |
| EHR alerts | Rowe 2023 | Assess for readiness and identify barriers and facilitators, Conduct local needs assessment |  |  |  |  | Remind clinicians |  |  | Change record systems | 4 |
| EHR alerts | Shelton 2015 |  |  |  | Use advisory boards and workgroups |  | Remind clinicians |  |  | Change record systems | 3 |
| EHR alerts | Singhal 2022 |  |  |  |  |  | Remind clinicians |  |  | Change record systems | 2 |
| EHR alerts | Tamblyn 2003 |  |  |  |  |  | Remind clinicians |  |  | Change record systems | 2 |
| EHR alerts | Tamblyn 2008 |  |  |  |  |  | Remind clinicians |  |  | Change record systems | 2 |
| EHR alerts | Walsh 2016 |  |  |  |  |  | Remind clinicians |  |  | Change record systems | 2 |
| EHR alerts | Wessell 2013 |  |  | Tailor strategies | Identify and prepare champions | Conduct educational meetings, Conduct educational outreach visits | Remind clinicians |  |  | Change record systems | 6 |
| EHR alerts | Mann 2020 |  |  | Tailor strategies | Involve executive boards | Conduct educational meetings, Distribute educational materials |  |  |  | Change record systems | 5 |
| Order sets and preference lists | Ancker 2021 |  |  |  |  |  |  |  |  | Change record systems | 1 |
| Order sets and preference lists | Buehrle 2020 | Audit and provide feedback, Conduct local needs assessment |  |  |  | Conduct educational meetings |  |  |  | Change record systems | 4 |
| Order sets and preference lists | Khadadah 2022 |  |  |  |  | Conduct educational meetings, Distribute educational materials, Work with educational institutions | Remind clinicians |  |  | Change record systems | 5 |
| Order sets and preference lists | Lin 2020 |  |  |  |  | Conduct educational meetings, Conduct ongoing training | Remind clinicians |  |  | Change record systems | 4 |
| Order sets and preference lists | Martins 2017 |  |  |  |  |  | Remind clinicians |  |  | Change record systems | 2 |
| Order sets and preference lists | Matulis 2017 |  |  |  |  | Conduct educational meetings | Create new clinical teams |  |  | Change record systems | 3 |
| Order sets and preference lists | Rozario 2020 |  |  |  | Involve executive boards | Develop educational materials, Distribute educational materials |  |  |  | Change record systems | 4 |
| Order sets and preference lists | Seppänen 2016 | Audit and provide feedback |  |  |  | Conduct educational meetings |  |  |  | Change record systems | 3 |
| Order sets and preference lists | vanWijk 2001 |  |  |  |  |  |  |  |  | Change record systems | 1 |
| Order sets and preference lists | Vardy 2005 | Conduct local needs assessment |  |  | Conduct local consensus discussions, Involve executive boards | Conduct educational meetings |  |  |  | Change record systems | 5 |
| Order sets and preference lists | Singh-Franco 2022 |  |  |  |  | Distribute educational materials | Remind clinicians | Involve patients/consumers and family members |  | Change record systems | 4 |
| Order sets and preference lists | Mann 2020 |  |  | Tailor strategies | Involve executive boards | Conduct educational meetings, Distribute educational materials |  |  |  | Change record systems | 5 |
| Order sets and preference lists | McCormick 2020 | Conduct local needs assessment |  |  |  | Conduct educational meetings | Remind clinicians |  |  | Change record systems | 4 |
