# Supplementary material for: The role of electronic health records systems in de-implementing low-value care in primary care: a scoping review
Source: Implement Sci Commun. 2025 Dec 19;6:138. doi: 10.1186/s43058-025-00826-6 (PMC12717702; doi:10.1186/s43058-025-00826-6)
Supplement: Supplementary file 6 — Additional file 6. [file 43058_2025_826_MOESM6_ESM.docx]

| **EHR Intervention Type** | **Type of De-Implementation Outcome** | Measurement Approaches | Findings | **Citations** |
| --- | --- | --- | --- | --- |
| EHR Alerts | Acceptability | Open-ended survey question on perceived effectiveness of intervention components | Mixed acceptability: Some felt it supported evidence-based prescribing while others felt it threatened clinical autonomy or did not perceive the need that they had to change | Ackerman et al., 2013 |
|  |  | Semi-structured interviews | Mixed acceptability: Some clinicians thought the alerts were useful as reminders while others felt that the alerts used outdated data and produced erroneous or irrelevant recommendations | Alagiakrishnan et al., 2016 |
|  |  |  | Some clinicians felt that they would be unwilling to change their practice patterns due to concerns on additional workload and threats to clinical autonomy. Others also felt that they do not overprescribe and do not need the alerts. Others also felt they did not trust the available evidence base to warrant changes. Almost 33% of clinicians reported the alerts had no impact on their prescribing.  Clinicians also reported high acceptability of the webinar that provided education on the alerts. | Gulliford et al., 2019 |
|  |  |  | Mixed acceptability: Some clinicians thought the alerts were useful, especially for novice clinicians, while others did not feel they were helpful because they believed they already followed guidelines | McDermott et al., 2014 |
|  |  | Informally collected feedback | Physicians were very receptive to having pharmacists review alerts and make medications recommendations for physicians to review | Cossette et al., 2019 |
|  |  | Closed-ended survey question | 30% of clinicians thought that the alert could be helpful for patient care | Gill et al., 2011 |
|  |  |  | Satisfaction with various components of alert ranged from 61.5% to 80.8% | Gulliford et al., 2019 |
|  |  |  | Mixed acceptability: The likelihood to recommend the alerts to others varied by type - prostate-specific antigens (67%), urinalysis or urine culture (38%), and diabetes (88%) | Rowe et al., 2023 |
|  | Adoption | Open-ended survey question on perceived effectiveness of intervention components | Mixed adoption: Those who thought the intervention might benefit them or could see the need to address overprescribing were likelier to use the intervention. Those who felt their clinical autonomy should not be challenged or thought they did not need to change were less likely to use the intervention | Ackerman et al., 2013 |
|  |  | Semi-structured interviews | 87% of clinicians reported intent to continue to use the tool | Alagiakrishnan et al., 2016 |
|  |  |  | Some clinicians felt that they would be unwilling to change their practice patterns due to concerns on additional workload | Gulliford et al., 2019 |
|  |  | Closed-ended survey question | 23% of intervention clinicians reported intent to continue to use the tool after the study ends | Gill et al., 2011 |
|  |  | Authors' observations | 94% of practices committed to using the alerts | Mann et al., 2020 |
|  |  |  | 74% of approached clinicians agreed to participate in the study | Persell et al., 2016 |
|  |  |  | 28.9% of physicians agreed to participate | Tamblyn et al., 2003 |
|  | Appropriateness | Open-ended survey question on perceived effectiveness of intervention components | Low appropriateness if the alerts cannot help address patients' expectations to receive medications or if the guidelines displayed are felt to not factor in the patient's medical complexity | Ackerman et al., 2013 |
|  |  | Semi-structured interviews | 87% of clinicians thought the alerts were available at appropriate points of the workflow. Clinicians also felt that alerts might fire in incorrect situations due to medication histories that were not reconciled | Alagiakrishnan et al., 2016 |
|  |  | Clinician review of alerts | 41.5% were felt to be clinically relevant | Cossette et al., 2019 |
|  |  | Closed-ended survey question | 25.9% of clinicians felt that the occurred at incorrect times of the visit | McDermott et al., 2014 |
|  |  |  | Most clinicians thought the alerts for prostate-specific antigens (67%), urinalysis or urine culture (88%), and diabetes (100%) were clinically accurate. They also thought the alerts for prostate-specific antigens (100%), urinalysis or urine culture (63%), and diabetes (88%) were fired appropriately. | Rowe et al., 2023 |
|  | Costs | Proxied costs saved based on Medicare reimbursement data | $51,538 cost savings in a six-month period | Anderson et al., 2020 |
|  |  | Proxied costs saved based on costs of providing care | Although no itemized breakdown exists, de-implementation costs included having vendor design the alerts, staff time needed to design and implement webinar and print materials.  Annual total costs estimated to be 1052£ for control arm and 1001£ for intervention arm. | Gulliford et al., 2019 |
|  | Feasibility | Open-ended survey question on perceived effectiveness of intervention components | Low feasibility for those who felt the tool was difficult to use or had too much information | Ackerman et al., 2013 |
|  |  | Semi-structured interviews | 50% of clinicians thought the alerts were disruptive to their workflows. Clinicians also expressed concerns that the alerts did not provide explicit details on how to discontinue drugs, monitoring for side effects, or acceptable alternatives to the de-implemented practice. They also hesitated to discontinue orders placed by other clinicians. Some clinicians also felt that those who only used the EHR after the patient visits may not benefit from alerts | Alagiakrishnan et al., 2016 |
|  |  |  | Mixed perceptions of usability: Clinicians felt the alerts were easy to use but felt there was little time to read through them and that they were not very salient | McDermott et al., 2014 |
|  |  | Closed-ended survey question | 44% of clinicians felt that the tool was disruptive to their workflows | Gill et al., 2011 |
|  |  |  | Most physicians who recalled seeing the alerts for prostate-specific antigens (67%), urinalysis or urine culture (50%), and diabetes (88%) reported that they were able to integrate the alerts into clinical practice.  There were mixed responses on to whether physicians thought that alerts for prostate-specific antigens (67%), urinalysis or urine culture (13%), and diabetes (75%) were not time-consuming. | Rowe et al., 2023 |
|  | Fidelity | Semi-structured interviews | 50% of clinicians thought the alerts appeared when they should not have. 87% felt there were situations where the alerts should have appeared but did not. Clinicians also expressed concerns that the alerts used outdated data (e.g., unreconciled medication history) and made irrelevant recommendations | Alagiakrishnan et al., 2016 |
|  |  | System logs | 25% of alerts were acknowledged and resulted in no changes to treatment. 15% of alerts resulted in clinicians accessing evidence or education resources. | Alagiakrishnan et al., 2016 |
|  |  |  | Alerts did not fire correctly for 202 patients because of missing information in the EHR. Furthermore, medical assistants were to respond to the alerts by showing a video to patients. This was done only 4.7% of the time. | Campbell et al., 2021 |
|  |  |  | At least 69% of intervention clinicians completed the training associated with intervention | Gill et al., 2011 |
|  |  |  | Across the sites, between <1% and 28% of eligible visits involved the clinician viewing the tool | Gulliford et al., 2019 |
|  |  |  | Across the sites, use of the calculator within the alerts ranged from 2.5% to 9.6% across eligible visits | Mann et al., 2020 |
|  |  |  | Alerts had fired for 57% of visits relating to prostate-specific antigen testing, 93% for visits relating urinalysis or urine cultures, and 100% for visits relating to diabetes | Rowe et al., 2023 |
|  |  | Closed-ended survey question | 55.6% of clinicians reported seeing the alerts during visits | McDermott et al., 2014 |
|  | Penetration | Closed-ended survey question | 51% of intervention clinicians reported use of the tool | Gill et al., 2011 |
|  |  | System logs | Alerts were not heavily used | Gonzales et al., 2013 |
|  |  |  | Across the sites, between <1% and 28% of eligible visits involved the clinician viewing the tool | Gulliford et al., 2019 |
|  |  |  | The tool was used in 39.7% of encounters | Hingorani et al., 2015 |
|  | Sustainability | System logs | The use of the calculator in the alert decreased from 16.4% to 2.7% over a time period of 33 months | Mann et al., 2020 |
| Order Sets and Preference Lists | Acceptability | Not assessed | Not assessed |  |
|  | Adoption | Not assessed | Not assessed |  |
|  | Appropriateness | Not assessed | Not assessed |  |
|  | Costs | Proxied cost savings based on costs of providing care | $124,380 CAD per year | Khadadah et al., 2022 |
|  |  | Proxied cost savings based on reimbursement payments received from Medicare | $1,060,640 USD per year | Rozario et al., 2020 |
|  | Feasibility | Not assessed | Not assessed |  |
|  | Fidelity | System logs | Across the sites, the acceptance of the order set ranged from 0.1% to 1.3% | Mann et al., 2020 |
|  | Penetration | Not assessed | Not assessed |  |
|  | Sustainability | Not assessed | Not assessed |  |
| Documentation Templates | Acceptability | Semi-structured interviews | Some clinicians thought the tool improved visit efficiency | Litvin et al., 2012 |
|  | Adoption | Authors' observations | Initially, not all clinicians wanted to adopt the tool but eventually they all committed to using it after the study team responded with having one-on-one meetings with non-committing physicians | Litvin et al., 2012 |
|  |  | Authors' observations | All clinicians agreed to use the tool | Mainous et al., 2013 |
|  | Appropriateness | Semi-structured interviews | Some clinicians thought the guidelines did not apply to all of their patients | Litvin et al., 2012 |
|  |  | Anecdotal feedback | Some clinicians disagreed with the guidelines | Litvin et al., 2013 |
|  | Costs | Not assessed | Not assessed |  |
|  | Feasibility | Semi-structured interviews | Clinicians felt the tool was difficult to use and struggled if there was no technical support or could not make modifications ot the template | Litvin et al., 2012 |
|  |  | Anecdotal feedback | Clinicians felt that tool use was impeded by patients demanding medications or concerns on missing diagnoses | Litvin et al., 2013 |
|  | Fidelity | Semi-structured interviews | If there are no computers in the exam room, the tool will be used before or after the visit rather than during the visit | Litvin et al., 2012 |
|  | Penetration | System logs | Across the practices, tool use was between 39.4% to 77.2% | Litvin et al., 2012 |
|  |  |  | Across the practices, template use ranged from 0% to 68.5% | Mainous et al., 2013 |
|  | Sustainability | Not assessed | Not assessed |  |
| Communication Tools Among the Care Team | Acceptability | Semi-structured interviews | Physicians appreciated having support from other care team members to help deprescribe | Lagisetty et al., 2020 |
|  | Adoption | Not assessed | Not assessed |  |
|  | Appropriateness | Direct feedback | Clinicians felt that some patients who have poor relationships with the clinician or would behave distrustfully towards involving other care team members would not be suitable for deprescribing efforts | Lagisetty et al., 2020 |
|  | Costs | Not assessed | Not assessed |  |
|  | Feasibility | Semi-structured interviews | Intervention was similar in design to those already used for chronic care management, which improved intervention uptake among clinicians | Lagisetty et al., 2020 |
|  | Fidelity | Not assessed | Not assessed |  |
|  | Penetration | Not assessed | Not assessed |  |
|  | Sustainability | Semi-structured interviews | There were concerns on whether the program could be sustained because it relied on pharmacist staffing levels | Lagisetty et al., 2020 |
|  |  | Authors' observations | Some sites hired additional nurses to try to sustain the intervention | Liebschutz et al., 2017 |
